# Double impact of cigarette smoke and mechanical ventilation on the alveolar epithelial type II cell

**DOI:** 10.1186/cc13795

**Published:** 2014-03-25

**Authors:** Jan Hirsch, Robert J Chalkley, Trevor Bentley, Alma L Burlingame, James A Frank

**Affiliations:** 1 Anesthesia and Perioperative Care, University of California, San Francisco VA Medical Center, San Francisco, CA, USA; 2 Anesthesia Service, San Francisco VA Medical Center, 4150 Clement Street, San Francisco, CA 94121, USA; 3 Mass Spectrometry Facility, Department of Pharmaceutical Chemistry, University of California, San Francisco, Genentech Hall, N472A, MC 2240, 600 16th Street, San Francisco, CA 94158-2517, USA; 4 Department of Medicine, University of California, San Francisco, and San Francisco VA Medical Center, San Francisco, CA, USA; 5 Pulmonary Division, Medicine Service, San Francisco VA Medical Center, 4150 Clement Street, San Francisco, CA 94121, USA

## Abstract

**Introduction:**

Ventilator-induced lung injury (VILI) impacts clinical outcomes in acute respiratory distress syndrome (ARDS), which is characterized by neutrophil-mediated inflammation and loss of alveolar barrier function. Recent epidemiological studies suggest that smoking may be a risk factor for the development of ARDS. Because alveolar type II cells are central to maintaining the alveolar epithelial barrier during oxidative stress, mediated in part by neutrophilic inflammation and mechanical ventilation, we hypothesized that exposure to cigarette smoke and mechanical strain have interactive effects leading to the activation of and damage to alveolar type II cells.

**Methods:**

To determine if cigarette smoke increases susceptibility to VILI *in vivo*, a clinically relevant rat model was established. Rats were exposed to three research cigarettes per day for two weeks. After this period, some rats were mechanically ventilated for 4 hours. Bronchoalveolar lavage (BAL) and differential cell count was done and alveolar type II cells were isolated. Proteomic analysis was performed on the isolated alveolar type II cells to discover alterations in cellular pathways at the protein level that might contribute to injury. Effects on levels of proteins in pathways associated with innate immunity, oxidative stress and apoptosis were evaluated in alveolar type II cell lysates by enzyme-linked immunosorbent assay. Statistical comparisons were performed by *t*-tests, and the results were corrected for multiple comparisons using the false discovery rate.

**Results:**

Tobacco smoke exposure increased airspace neutrophil influx in response to mechanical ventilation. The combined exposure to cigarette smoke and mechanical ventilation significantly increased BAL neutrophil count and protein content. Neutrophils were significantly higher after smoke exposure and ventilation than after ventilation alone. DNA fragments were significantly elevated in alveolar type II cells. Smoke exposure did not significantly alter other protein-level markers of cell activation, including Toll-like receptor 4; caspases 3, 8 and 9; and heat shock protein 70.

**Conclusions:**

Cigarette smoke exposure may impact ventilator-associated alveolar epithelial injury by augmenting neutrophil influx. We found that cigarette smoke had less effect on other pathways previously associated with VILI, including innate immunity, oxidative stress and apoptosis.

## Introduction

Tobacco use, primarily in the form of cigarettes, and exposure to tobacco smoke pollution have caused the premature deaths of more than 14 million Americans since 1964 [1]. Although per capita consumption of tobacco products has declined substantially since 1950, tobacco smoke remains a significant contributor to adverse health outcomes all over the world [2]. Cigarette smoke exposure contributes to a variety of chronic lung diseases, but less is known about the possible contribution of smoking to acute respiratory distress syndrome (ARDS). Recent epidemiological data suggest that smoking may be a risk factor affecting the incidence of acute lung injury and the prognosis patients with acute lung injury [3-6] and other acute respiratory complications [7,8], both in the ICU [4,5] and in the perioperative setting [6-9]. The authors of one large epidemiologic study [4] suggested this relationship with evidence of a dose–response effect between cigarette smoking and ARDS, including approximately 50% of ARDS cases associated with cigarette smoking. However, in a large study of 5,584 patients by Gajic and coworkers [10], past or current smoking combined was not a statistically significant risk factor for the development of acute lung injury (*P* < 0.07). Therefore, additional data are needed to substantiate the possibility that smoke exposure alters alveolar epithelial cell function and predisposes cells to additional injury. Previously published experimental data show that lesions induced in rat lung by chronic tobacco smoke inhalation include alveolar type II (ATII) cell hyperplasia [11]. A direct inhibitory effect of cigarette smoke constituents on surfactant secretion in ATII cells has also been demonstrated [12]. Both cigarette smoke and mechanical ventilation have been associated with increased oxidative stress in the lung epithelium. Therefore, distal lung epithelial dysfunction may be a potential contributor to the increased incidence of ARDS in smokers.

Over the past decade, it has been established that mechanical ventilation can be a major contributor to the prognosis of ARDS patients [13], and substantial headway has been made in understanding the pathophysiology of this disease [14]. We previously described the impact of mechanical ventilation with different tidal volumes on the proteome of ATII cells in a rat model [15]. On the basis of these data, we postulated that smoke exposure and mechanical strain due to mechanical ventilation may have interactive effects leading to oxidative stress, inflammation and epithelial barrier dysfunction in association with changes in the ATII cell proteome.

We hypothesized that rats exposed to mainstream cigarette smoke would develop more severe ventilator-induced lung injury (VILI) compared to nonexposed animals. We further predicted that this susceptibility would be reflected in measurable changes in ATII cell protein content. Therefore, we performed ATII cell isolation followed by labeling, multidimensional chromatographic fractionation and isobaric tag for relative and absolute quantitation liquid chromatography tandem mass spectrometry (iTRAQ LC-MS/MS) to discover markers for cellular pathways of activation and injury and followed up on the results using more sensitive immunological methods.

## Materials and methods

### Animal model

All animal experiments were carried out at the research laboratories of the San Francisco Veterans Affairs Medical Center with the approval of the local ethics committee, the San Francisco Veterans Affairs Committee on Animal Research (Institutional Animal Care and Use Committee 07-062-01). We cared for the animals in accordance with the National Institutes of Health (NIH) guidelines for ethical research (NIH Publication 80-123, revised 1985). Inbred male Sprague-Dawley rats (Harlan Laboratories, Indianapolis, IN, USA) were used for this study.

To determine if cigarette smoke increases susceptibility to VILI, a rat model was established. A total of 82 male Sprague-Dawley rats were assigned to four groups by convenience sampling. Limitations in samples that could be obtained from one rat and the amounts that were necessary to perform the assays made it necessary to perform experiments not in the same rats, but in identically treated animals.

Two groups of rats were exposed to the mainstream smoke of three University of Kentucky 3R4F research cigarettes per day over the course of 30 minutes, on 5 days weekly, for 2 weeks (S) in a previously described smoke exposure chamber [16]. The experimental setup is displayed in Additional file 1. Smoke exposure was titrated in preliminary experiments to a carboxyhemoglobin level between 5% and 10% measured 30 minutes after exposure. It is noteworthy, however, that carboxyhemoglobin levels may vary significantly [17]. Two other groups of rats were exposed to room air only (NS). At the end of the 2-week smoke exposure period, one-half of the smoke-exposed and control rats were anesthetized and placed on mechanical ventilation (tidal volume 10 ml/kg, positive end-expiratory pressure (PEEP) 4 cm H_2_O) (V)). The ventilation settings were chosen to match the results of a survey on ICU ventilator settings after the ARDS Network trial [18]. These settings are known to induce a mild degree of lung injury in rats. Rats were anesthetized with pentobarbital and mechanically ventilated for 4 hours at 21% fraction of inspired oxygen using a rodent ventilator (Harvard Apparatus, Holliston, MA, USA) [19]. The goals in administering anesthesia were to avoid use of medication that might potentially change the results (ketamine → secretion and bronchodilatation; anesthetic gases → bronchodilatation) and to maintain ethical conduct by preventing awareness by ensuring deep anesthesia without paralytics while constantly monitoring of ventilation parameters. Anesthesia was confirmed by assessing movement and heart rate response to paw pinch in 30-minute intervals, with response triggering an additional intraperitoneal injection of pentobarbital. Ventilation was monitored by constant monitoring and recording of ventilation pressures and volumes. Rats were given subcutaneous injections of sterile normal saline (3 ml) every 2 hours for volume replacement. The respiratory rate was established in preliminary experiments using arterial blood gas samples to obtain normal pH and partial pressure of carbon dioxide in arterial blood values. The remaining rats did not undergo mechanical ventilation (NV).

At the end of the experiments, anesthetized rats were killed by transection of the abdominal aorta. Bronchoalveolar lavage (BAL) was performed in all animals with 7 ml of normal saline. ATII cells were isolated from the lungs of all of the rats as previously described [20] by digesting lung tissue with elastase and panning the resultant cell suspension on plates coated with immunoglobulin G to deplete other cell types, such as macrophages. After the panning and washing steps, the recovered cells were checked for purity and viability microscopically using the trypan blue exclusion method.

#### Cell lysis and enrichment of subcellular fractions

Immediately after isolation, cells were counted and viability was evaluated under the microscope using a hemocytometer and the trypan blue exclusion method. We observed no significant differences in cell count or viability between the different groups. Overall viability was consistently above 95% in all experiments. Cells were then collected by centrifugation (6 minutes at 1,000 × *g*) and rinsed in phosphate-buffered saline. The cells were resuspended in ice-cold lysis buffer and washed two times by centrifugation and resuspension in lysis buffer. The lysis buffer consisted of ice-cold isoosmolar ammonium bicarbonate solution containing 2 mM ethylenediaminetetraacetic acid, 2 mM ethylene glycol tetraacetic acid, complete protease inhibitor cocktail (Sigma P2714; Sigma-Aldrich, St Louis, MO, USA) and antiphosphatases (Sigma P2850 and P5726; Sigma-Aldrich). Cell lysis was performed on ice by short bursts of sonication (approximately 1 to 4 seconds) at the lowest setting of the sonicator under visual control with the microscope until the cell membranes were broken up while the majority of the nuclei were still intact.

The method of enrichment of subcellular fractions was based on previous work [21-24]. Isolated ATII cells were centrifuged at 1,000 average relative centrifugal force for 10 minutes to separate the nuclear fraction from the postnuclear supernatant. The supernatant was centrifuged at an average of 10,000 × *g* for 10 minutes to bring down the mitochondrial fraction, which was stored at -80°C. The postmitochondrial supernatant was centrifuged at an average of 50,000 × *g* for 100 minutes (5 × 10^6^*g*/min) at 4°C to separate the membrane microsomal fraction from the cytosolic fraction. The resulting fractions were frozen at -80°C for later analysis.

### Mass spectrometry analysis

Samples taken from three rats from each group were evaluated in three independent iTRAQ comparisons and cation exchange chromatography runs. iTRAQ labeling was performed using commercially available reagents (AB SCIEX, Framingham, MA, USA) based on the method described by Ross *et al*. [25]. Samples were purified by acetone precipitation as described previously [26]. Twenty microliters of 100 mM triethylammonium bicarbonate dissolution buffer (AB SCIEX) and 1 μl of 0.05% w/v SDS solution were added to each of the sample tubes containing 125 μg of protein. The tubes were vortexed, 2 mM Tris-(2-carboxyethyl) phosphine reducing reagent (AB SCIEX) were added and the tubes were incubated at 60°C for 1 hour. The tubes were spun, 1 μl of cysteine blocking reagent (AB SCIEX) was added and the tubes were incubated for 10 minutes at room temperature. Tryptic digestion was initiated by the addition of 2% w/w side-chain-modified, tosyl phenylalanyl chloromethyl ketone–treated porcine trypsin in double-distilled water (ddH_2_O)). The reaction was allowed to proceed at 37°C for 12 hours. The tubes were spun, iTRAQ reagent (AB SCIEX) dissolved in 70 μl of ethanol was added to the tube and the samples were incubated for 1 hour at room temperature. The reaction was then quenched with 30 μl of ddH_2_O. Labeling was checked by running an aliquot of the samples on an QSTAR electrospray time of flight mass spectrometer (AB SCIEX, Framingham, MA) after purification with ZipTip C_18_ reversed-phase (RP) columns (EMD Millipore, Danvers, MA, USA). A 5% fraction of the pooled samples was run in one initial RP and one MS run. The overall the stable isotope peak intensities were then compared to check for systematic errors in labeling and protein concentration. Subsequently, the differentially labeled ischemia-treated and control samples were pooled. Each of the quadruplets was then fractionated into 16 different fractions by cation exchange chromatography. Cation exchange chromatography was performed using an Äkta system (GE Healthcare Life Sciences, Piscataway, NJ, USA) with 35 nl of ultraviolet-visible cell and a 1,000 μl injection loop). We used a 2.0- × 10-mm PolySULFOETHYL A column (PolyLC, Columbia, MD, USA) at a 200 μL/min flow rate. For solvent A, 25% acetonitrile and 0.075% formic acid (FA) and were used, and for solvent B, solvent A and an additional 400 mM ammonium chloride were used. A gradient of 0% solvent B, 0 to 10 minutes, and 0% to 50% solvent B, 3 to 60 minutes, was set. Fractions were collected from the mixture. The samples were then dried in the SpeedVac concentrator (Thermo Scientific, Pittsburgh, PA, USA) and resuspended in 0.1% v/v FA. The fractions were desalted in an automated fashion using the Äkta system with a Jupiter high-performance liquid chromatography C-_18_ column (Phenomenex, Torrance, CA, USA).

### Mass spectrometry measurements

Six fractions from each sample were further analyzed, resulting in 72 fractions (4 groups × 3 samples × 6 fractions). These samples were further separated by RP chromatography. The separation of the peptides was achieved by a gradient of increasing acetonitrile in water over 60 minutes using 0.1% v/v FA as the ion-pairing agent on a 75-μm ID Jupiter Proteo RP column (Phenomenex). Tryptic peptides were analyzed by LC-MS/MS on a QSTAR electrospray mass spectrometer (AB SCIEX) operating in positive ion mode and connected in line with the chromatography unit as described elsewhere [27]. Samples were separated by nano–liquid chromatography using a flow rate of 300 nl/min.

### Individual protein content and activity measurements

The following commercially available assays were used according to the manufacturers’ instructions: rat interleukin 1β (IL-1β) (ER2ILB; (Thermo Scientific); catalase assay kit (219265; Calbiochem, San Diego, CA, USA); Assay Designs caspase 3 assay (907-013), heat shock protein 70 (HSP70) (EKS 700B), caspase 8 (850-221-K101) and caspase 9 (850-223-K101) (all from Enzo Life Sciences, Farmingdale, NY, USA); cell death detection enzyme-linked immunosorbent assay (ELISA) (11774425001; Roche Applied Science, Indianapolis, IN, USA); nuclear factor κB p65 (NFκB/p65) (IMK-503 (Imgenex, San Diego, CA, USA); and rat Toll-like receptor 4 Rat TLR-4 # E0753R (USCNL Life, Wuhan, China). Protein content was determined using the Protein DC assay kit (500-0111; Bio-Rad Laboratories, Hercules, CA, USA). The protein data for caspase 3 and HSP70 were determined by ELISA in both the nuclear and cytosolic fractions. Although there were differences in total protein amount with enrichment, there were identical protein patterns in both fractions (data not shown). The choices of data points per experiment were made based on the availability of equal numbers of specimens and the size of the available assays.

### Data analysis

The 72 peak lists from MS experiments were combined and interrogated against the UniProt/SwissProt *Rattus norvegicus* protein database [PR:P06761] using ProteinProspector 5.10 software package (University of California, San Francisco; [28-30]). A minimal ProteinProspector score of 22, a minimal peptide score of 15, a minimal discriminant score of 0 and a maximal expectation value of 1E-2 were used for identification criteria (see online supplement). In order to estimate the false discovery rate (FDR) for the results, the same search was performed against a concatenated database of normal and decoy sequences, assuming that for every match to the decoy database at the employed acceptance criteria, there would also be an incorrect match among the normal database sequences. Protein identification was cross-checked manually. Quantification was determined by calculating the ratios of the areas under the reporter peaks using the Search Compare tool in the ProteinProspector suite of programs. For each identified protein, we calculated the means and standard deviations of the peak intensity ratios from all peptides assigned to a given protein.

The results of the ELISA and BAL measurements were compared by performing *t*-tests for independent samples using SPSS version 16.0 for Windows software (IBM SPSS, Chicago, IL, USA). A Kolmogorov-Smirnov goodness-of-fit test was performed against a normal distribution. There was no significant difference from a normal distribution, with the exception of some groups of basophil and eosinophil percentage data, for which no statistical tests were performed. Therefore, the data herein are described as means and standard deviations, with parametric statistical tests used for comparisons. A Levene test for homogeneity of variance was used in all samples to test for heteroscedasticity, and *P*-values were corrected accordingly using the algorithm in SPSS. Because heteroscedasticity resilient to transformation precluded the use of analysis of variance, a total of 22 *t*-tests were performed and *P*-value thresholds were established to correct for multiple comparisons as described by Benjamini and Hochberg [31] using the script published by IBM for SPSS software [32]. *P*-values and FDR threshold values are provided in Table 1.

**Table 1:** ***P*-values obtained from statistical comparisons between the nonsmoke/nonventilated, ventilation-alone and mechanical ventilation groups**^**a**^

**Groups compared**	**Measurement**	***P*-value**	**FDR rank**	**FDR threshold *P*-value**	**Significance after FDR correction**^**b**^
NSNV vs. SV	Total cell BAL	0.0006	1	0.0023	+
NSNV vs. SV	PMN cell total BAL	0.0013	2	0.0045	+
NSNV vs. SV	BAL Protein	0.0013	3	0.0068	+
NSNV vs. SV	Nuclear HSP70	0.0302	7	0.0159	-
NSNV vs. SV	Membrane TLR4	0.0392	9	0.0205	-
NSNV vs. SV	Cytosolic caspase 8	0.0502	10	0.0227	-
NSNV vs. SV	Nuclear NFκBp65	0.1151	13	0.0295	-
NSNV vs. SV	Cytosolic caspase 9	0.1167	14	0.0318	-
NSNV vs. SV	Nuclear caspase 3	0.1520	16	0.0364	-
NSNV vs. SV	Nuclear DNA fragments	0.3524	19	0.0432	-
NSNV vs. SV	Cytosolic catalase	0.7059	22	0.0500	-
NSV vs. SV	Nuclear DNA fragments	0.0082	4	0.0091	+
NSV vs. SV	PMN cell total BAL	0.0108	5	0.0114	+
NSV vs. SV	Membrane TLR4	0.0176	6	0.0136	-
NSV vs. SV	Cytosolic caspase 8	0.0352	8	0.0182	-
NSV vs. SV	BAL protein	0.0875	11	0.0250	-
NSV vs. SV	Nuclear NFκBp65	0.0897	12	0.0273	-
NSV vs. SV	Nuclear HSP70	0.1228	15	0.0341	-
NSV vs. SV	Nuclear caspase 3	0.1871	17	0.0386	-
NSV vs. SV	Cytosolic caspase 9	0.2767	18	0.0409	-
NSV vs. SV	Cytosolic catalase 8	0.4090	20	0.0455	-
NSV vs. SV	Total cells BAL	0.4549	21	0.0477	-

^a^BAL: Bronchoalveolar lavage; FDR: False discovery rate; HSP: Heat shock protein; NSNV: Nonsmoke/nonventilated; NSV: Ventilation alone; NFκB: Nuclear factor κB; PMN: Polymorphonuclear; SV: Mechanical ventilation; TLR4: Toll-like receptor 4. ^b^The right column indicates statistical significance after performing the FDR calculation (+ signifies statistical significance after correction and - means no statistical significance after correction).

## Results

### Bronchoalveolar lavage

#### Neutrophil cell count

**Figure 1:**
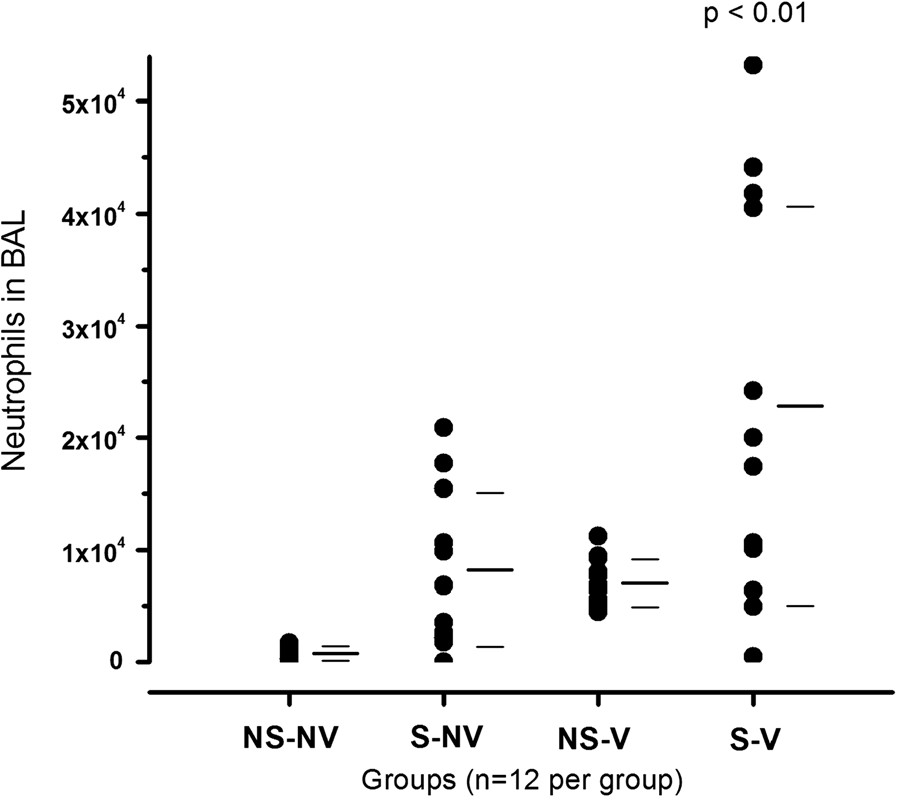
**Neutrophils in bronchoalveolar lavage fluid with and without exposure to smoke and mechanical ventilation.** The four groups depicted are nonsmoke/nonventilation (NS-NV), smoke/nonventilation (S-NV), nonsmoke/ventilation (NS-V) and smoke/ventilation (S-V). To the right of the dot blots, mean ± SD values are indicated by bars. The combination of the two stimuli resulted in a significant increase in polymorphonuclear cells (*P* < 0.01) compared to all other groups, including NS-V rats.

To determine the inflammatory response to smoke exposure and mechanical ventilation, the number of neutrophils in the BAL fluid (BALF) was measured. Analysis of cell percentages and overall numbers of different cell populations (Figure 1 and Table 2) demonstrated an increase in neutrophils after both smoke exposure and mechanical ventilation alone. The combination of both conditions resulted in a more-than-additive, statistically significant increase in neutrophils. There was a statistically significant difference between the BALF neutrophils from rats after ventilation alone and from rats after ventilation and smoke exposure combined (Table 1).

**Table 2:** **Cell differential in the bronchoalveolar lavage fluid**^**a**^

**Cell-type comparisons**	**NSNV**	**SNV**	**NSV**	**SV**
Differentials				
Macrophages (%)	94.33 ± 3.58	64.75 ± 25.54	79.42 ± 4.91	42.08 ± 38.50
PMN cells (%)	2.75 ± 1.91	22.67 ± 15.36	18.50 ± 3.85	55.58 ± 38.67
Lymphocytes (%)	1.58 ± 1.68	1.08 ± 1.08	1.08 ± 1.24	1.58 ± 2.94
Eosinophils (%)	1.25 ± 1.42	3.00 ± 5.48	0.67 ± 1.30	0.50 ± 0.80
Basophils (%)	0.08 ± 0.29	0.33 ± 0.65	0.33 ± 0.65	0.25 ± 0.45
Cell numbers				
Macrophages	2.32 × 10^6^ ± 9.48 × 10^5^	2.15 × 10^6^ ± 9.88 × 10^5^	3.18 × 10^6^ ± 1.37 × 10^6^	2.02 × 10^6^ ± 2.05 × 10^6^
PMN cells	7.74 × 10^4^ ± 6.40 × 10^4^	8.20 × 10^5^ ± 6.83 × 10^5^	7.05 × 10^5^ ± 2.12 × 10^5^	2.28 × 10^6^ ± 1.78 × 10^6^*^+^
Lymphocytes	5.07 × 10^4^ ± 6.00 × 10^4^	3.44 × 10^4^ ± 3.93 × 10^4^	3.81 × 10^4^ ± 3.74 × 10^4^	6.18 × 10^4^ ± 1.16 × 10^5^
Eosinophils	3.80 × 10^4^ ± 5.64 × 10^4^	1.40 × 10^5^ ± 3.24 × 10^5^	2.11 × 10^4^ ± 4.02 × 10^4^	2.04 × 10^4^ ± 3.08 × 10^4^
Basophils	2.10 × 10^3^ ± 7.27 × 10^3^	1.15 × 10^4^ ± 2.28 × 10^4^	1.41 × 10^4^ ± 2.71 × 10^4^	9.91 × 10^3^ ± 1.86 × 10^4^
Total cells	2.49 × 10^6^ ± 1.08 × 10^6^	3.36 × 10^6^ ± 1.25 × 10^6^	3.96 × 10^6^ ± 1.53 × 10^6^	4.40 × 10^6^ ± 1.25 × 10^6^*

^a^NSNV: Nonsmoke/nonventilated; NSV: Ventilation alone; PMN: Polymorphonuclear; SNV: Smoke/nonventilated; SV: Mechanical ventilation. Data presented are the cell differentials (percentage of cells) and the absolute cell numbers in the bronchoalveolar lavage for all cells combined and among different cell types. *P < 0.05 vs. NSNV; ^+^*P* < 0.05 vs. NSV.

#### Alveolar barrier permeability

**Figure 2:**
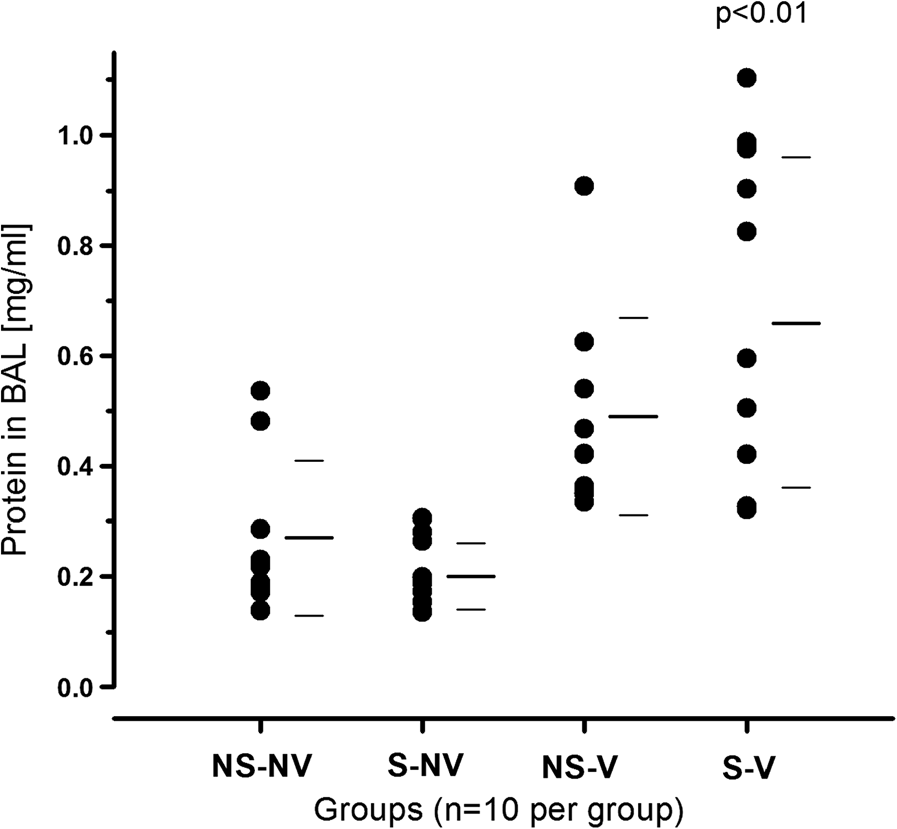
**Protein content in bronchoalveolar lavage fluid with and without exposure to smoke and mechanical ventilation.** Mechanical ventilation plus smoke exposure (S-V) led to significant increases in bronchiolar lavage (BAL) fluid protein content compared to normal controls (*P* < 0.01). Even though the *P*-value is less than 0.01, the addition of cigarette smoke exposure to ventilated rats did not lead to a significant further increase of BAL protein content after correction for false discovery rate (NSV vs. SV). The bars to the right of the data points indicate means ± SD. NS-NV: nonsmoke/nonventilated; NS-V: Ventilation alone; S-NV: Smoke/nonventilated.

To determine potential alterations in permeability to the alveolar–vascular barrier, we measured alveolar protein content in the BALF. After mechanical ventilation and smoke exposure combined, protein content in the BALF, a surrogate for macromolecular permeability, increased significantly (Figure 2 and Table 1), but there was no significant increase after mechanical ventilation alone. The average protein contents were as follows: NSNV = 0.27 ± 0.1435 mg/ml, SNV = 0.20 ± 0.06 mg/ml, NSV = 0.49 ± 0.18 mg/ml and SV = 0.66 ± 0.30 mg/ml.

#### Interleukin 1β enzyme-linked immunosorbent assay

There was no significant difference in the IL-1β content in BALF among the groups: NSNV = 68.20 ± 8.46 ng/ml, SNV = 67.88 ± 9.42 ng/ml, NSV = 62.97 ± 8.73 ng/ml and SV = 65.87 ± 10.93 ng/ml.

### Mass spectrometry analysis

MS data were obtained for a total of 186 proteins (see online supplement for a complete list). The total number of decoy proteins was 1, resulting in an estimated FDR of 0.53%. The resulting protein list was evaluated to discover vantage points for further analysis of the samples, and a list of 20 proteins of interest was compiled (see Table 3 and Additional file 2 for full qualitative and quantitative MS data).

**Table 3:** A selection of proteins of interest from the mass spectrometry analyses

**Accession no.**	**Protein name**	**MW**	**pI**
[PR:P04762]	Catalase	59,757.70	7.10
[PR:Q63716]	Peroxiredoxin 1	22,109.60	8.30
[PR:P35704]	Peroxiredoxin 2	21,783.90	5.30
[PR:O35244]	Peroxiredoxin 6	24,818.80	5.60
[PR:P08010]	Glutathione *S*-transferase μ2	25,702.90	6.90
[PR:P34058]	Heat shock protein (HSP)90β	83,281.90	5.00
[PR:P82995]	Heat shock protein HSP90α	84,815.60	4.90
[PR:P63018]	Heat shock cognate 71-kDa protein	70,871.60	5.40
[PR:P14659]	Heat shock-related 70-kDa protein 2	69,642.20	5.50
[PR:P55063]	Heat shock 70-kDa protein 1-like	70,549.80	5.90
[PR:Q07439]	Heat shock 70-kDa protein 1A/1B	70,185.90	5.60
[PR:P42930]	Heat shock protein β1	22,892.80	6.10
[PR:P26772]	10-kDa heat shock protein, mitochondrial	10,901.80	8.90
[PR:P63039]	60-kDa heat shock protein, mitochondrial	60,956.00	5.90
[PR:P62161]	Calmodulin	16,837.70	4.10
[PR:P18418]	Calreticulin	47,995.80	4.30
[PR:P67779]	Prohibitin	29,820.30	5.60
[PR:P00697]	Lysozyme C1	16,729.30	9.30
[PR:P22355]	Pulmonary surfactant-associated protein B	41,590.40	6.20

The UniProt/SwissProt accession numbers and protein names, molecular weight (MW) in daltons and isoelectric point (pI) are provided. A complete protein list is provided in the online supplement.

#### Oxidative pathways

We obtained quantitative information for the proteins catalase, peroxiredoxin 1, peroxiredoxin 2 and peroxiredoxin 6.

#### Heat shock proteins and mediators of apoptosis

Quantitative proteomics indicated content changes in HSP70 and calmodulin, the former being an inhibitor of apoptosis [33] and the latter being involved in proapoptotic pathways [34].

#### Innate immunity proteins

There was considerable variability in the content of lysozyme, an integral part of the innate immune response. In addition, pulmonary surfactant protein B has been shown to be potentially involved in innate immunity pathways [35].

### Immunochemical analysis

The results of our data measurements are provided in Table 4.

**Table 4:** **Results of immunochemical measurements in alveolar type II cells**^**a**^

**Cell type**	**NSNV**	**SNV**	**NSV**	**SV**
*Catalase,* nmol/min (*n* = 8 for each group)	3.40 × 10^-5^ ± 2.52 × 10^-5^	8.01 × 10^-6^ ± 9.03 × 10^-6^	8.93 × 10^-5^ ± 1.19 × 10^-4^	2.54^-5^ ± 5.72^-5^
*Toll-like receptor 4,* μg/cell (*n* = 8 for each group)	3.82 × 10^-7^ ± 2.92 × 10^-7^	1.81 × 10^-7^ ± 1.28 × 10^-7^	6.94 × 10^-7^ ± 5.17 × 10^-7^	1.07 × 10-^6^ ± 8.14 × 10^-7^
NFκBp65, ng/cell (*n* = 8 for each group)	4.20 × 10^-6^ ± 3.43 × 10^-6^	4.14 × 10^-6^ ± 1.89 × 10^-6^	5.08 × 10^-6^ ± 4.06 × 10^-6^	9.28 × 10^-6^ ± 7.25 × 10^-6^
*HSP70,* ng/cell (*n* = 8 for each group)	1.75 × 10^-7^ ± 1.05 × 10^-7^	5.24 × 10^-7^ ± 2.27 × 10^-7^	6.37 × 10^-7^ ± 5.23 × 10^-7^	1.24 × 10^-6^ ± 1.11 × 10^-6^
*Caspase 3,* U/cell (*n* = 10 for each group)	3.56 × 10^-4^ ± 3.22 × 10^-4^	4.14 × 10^-4^ ± 2.48 × 10^-4^	1.72 × 10^-4^ ± 1.11 × 10^-4^	1.01 × 10^-3^ ± 1.13 × 10^-3^
*Caspase 8,* U/cell (*n* = 7 for each group)	5.13 × 10^-8^ ± 2.71 × 10^-8^	7.42 × 10^-8^ ± 4.71 × 10^-8^	3.85 × 10^-8^ ± 1.38 × 10^-8^	1.76 × 10^-7^ ± 1.11 × 10^-7^
*Caspase 9,* U/cell (*n* = 7 for each group)	6.03 × 10^-8^ ± 3.71 × 10^-8^	1.65 × 10^-7^ ± 1.05 × 10^-7^	4.70 × 10^-8^ ± 1.39 × 10^-8^*	3.51 × 10^-7^ ± 4.20 × 10^-7^
*DNA fragments*, *% absorbance compared with NSNV* (*n* = 8 for each group)	100.00 ± 78.57	62.19 ± 108.49	15.73 ± 129.04	143.07 ± 69.43*

^a^HSP: Heat shock protein; NSNV: Nonsmoke/nonventilated; NSV: Ventilation alone; NFκB: Nuclear factor κB; SV: Mechanical ventilation. *P < 0.05 vs. NSNV; Data are means ± SD.

For nuclear DNA fragments, an indicator of apoptotic activity [36], a significantly higher content after smoke exposure with mechanical ventilation compared to mechanical ventilation alone was present (Table 1). There was no statistically significant difference between normal control rats and rats exposed to both smoke and ventilation. We used mouse monoclonal antibodies directed against DNA and histones in the assay to determine a potential endpoint of apoptotic activity.

Catalase activity was measured to determine antioxidant activity in the ATII cell cytoplasmic fraction [37]. When we compared normal controls with the ventilation-alone group, we found no significant difference after smoke exposure with or without mechanical ventilation (Table 1).

**Figure 3:**
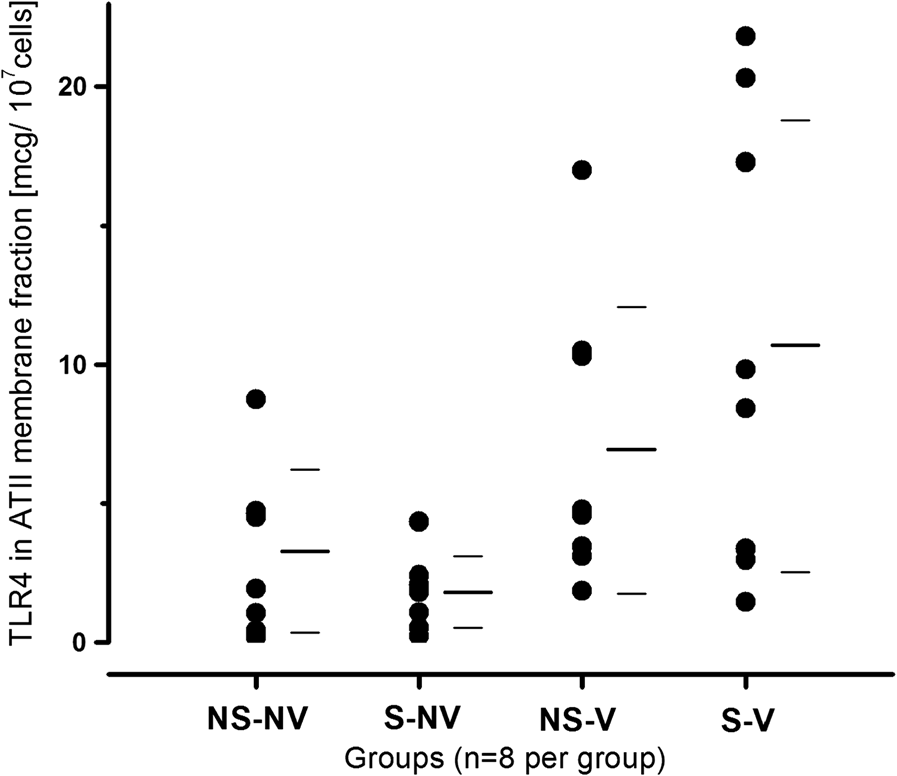
**Content in Toll-like receptor 4 in the membrane fraction of rat alveolar type II cells with or without exposure to smoke and mechanical ventilation.** Smoke exposure and mechanical ventilation alone did not lead to a significant increase in TLR4 compared to normal controls. The combination of the two stimuli resulted in a nonsignificant increase in TLR4 compared with both the nonsmoke/nonventilated (NS-NV) and smoke/nonventilated (S-NV) groups (both *P* < 0.05, not significant after correction for false discovery rate). ATII: alveolar type II cells; NS-V: Nonsmoke/ventilated; S-V: Smoke/ventilated. Bars represent mean and standard deviation of the data.

The cellular content of the lipopolysaccharide receptor TLR4 [38] was determined in the ATII cell membrane fraction to investigate innate immune activation via this pathway. Compared to control and ventilation-alone animals, the increase in TLR4 content was not significantly increased after smoke exposure and mechanical ventilation (Table 1 and Figure 3).

NFκBp65 was measured in the ATII cell nuclear fraction to determine activation of inflammatory and antioxidant activity via this pathway [39]. The cellular content in nonexposed rats was not significantly different from the content in smoke exposed and mechanically ventilated rats (Table 1). Compared to mechanical ventilation alone, there was no statistically significant change in cellular content after smoke exposure.

**Figure 4:**
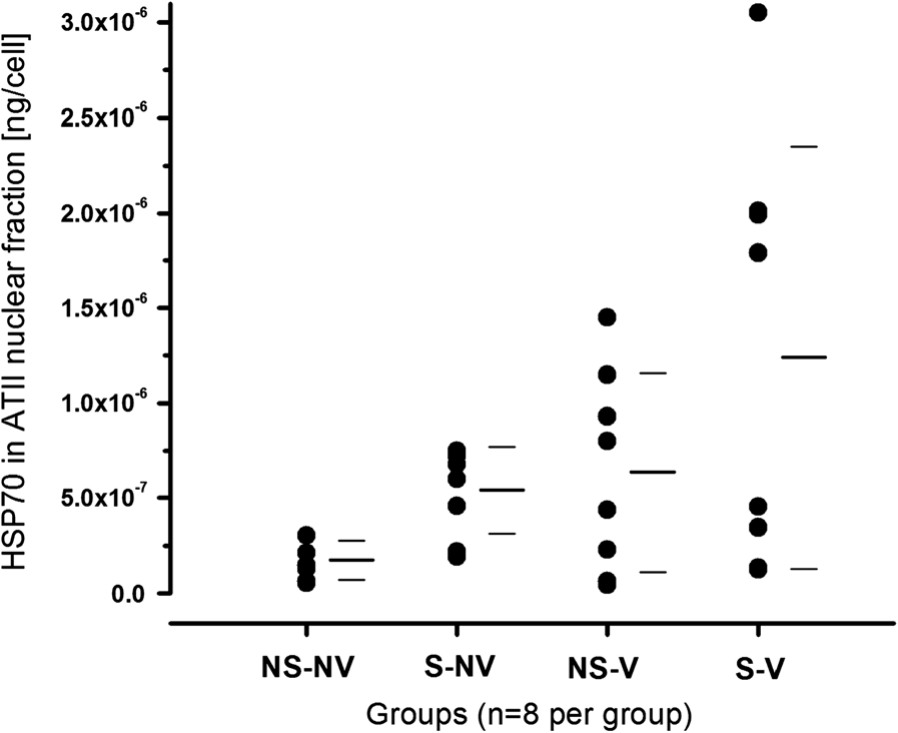
**Content in heat shock protein 70 in the nuclear fraction of rat alveolar type II cells with or without exposure to smoke and mechanical ventilation.** Smoke exposure and mechanical ventilation alone did not lead to an appreciable increase in Toll-like receptor 4. The combination of the two stimuli resulted in a nonsignificant increase in nuclear fraction heat shock protein 70 (HSP70) compared to both the nonsmoke/nonventilated (NS-NV) and the smoke/nonventilated (S-NV) groups. AVII: alveolar type II cells; NS-V: nonsmoke/ventilated; S-V: smoke/ventilated (*P* < 0.05 for NSNV vs. SV, not significant after correction for false discovery rate). Bars represent mean and standard deviation of the data.

HSP70 content was measured in the nuclear fraction to determine the activation state of the inflammasome of ATII cells, particularly in response to oxidative stress [40] (Figure 4). There was no significant difference between normal control rats and rats after smoke exposure and mechanical ventilation (Table 1) or between mechanical ventilation alone and smoke exposure and mechanical ventilation combined. A very similar distribution pattern was present for nonfractionated cells and the cytosolic fraction (data not shown).

**Figure 5:**
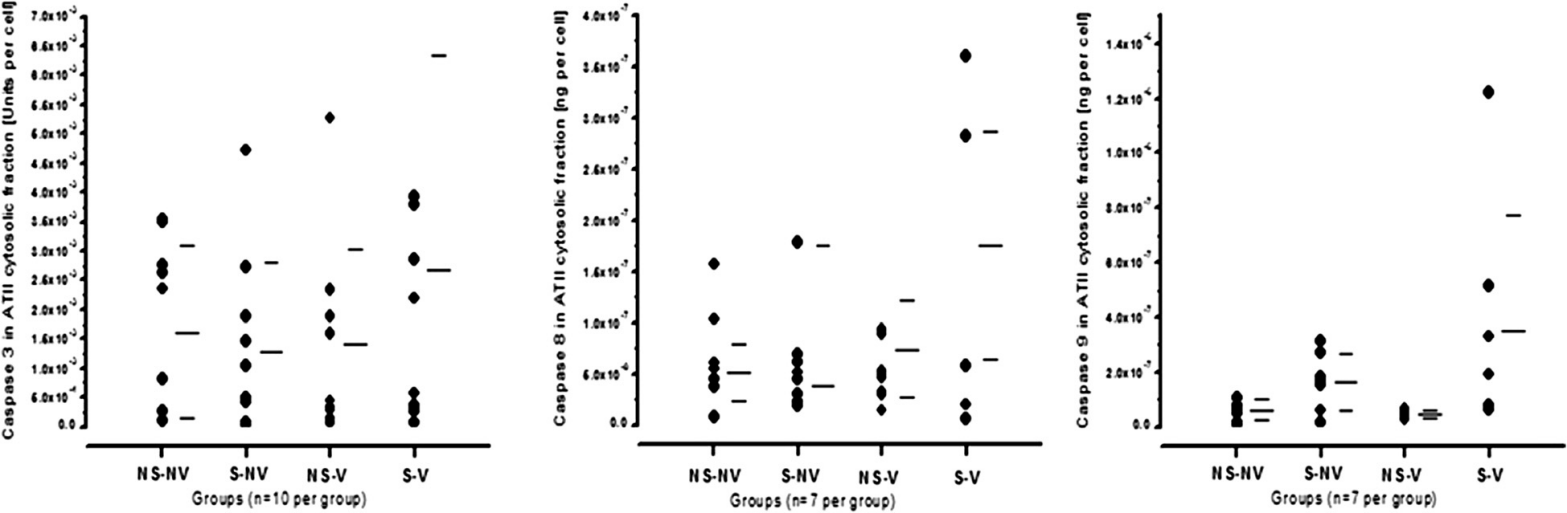
**Caspases 3, 8 and 9 activity in rat alveolar type II cells with or without exposure to smoke and mechanical ventilation.** Smoke exposure and mechanical ventilation alone did not lead to an increase. The combination of the two stimuli resulted in nonsignificant increases in caspase activity compared to all other groups. ATII: Alveolar type II cells; NS-NV: nonsmoke/nonventilated; NS-V: nonsmoke/ventilated; S-NV: smoke/nonventilated; S-V: smoke/ventilated. Bars represent mean and standard deviation of the data.

Caspases 3, 8 and 9 were measured to investigate potential contributions of their respective pathways of activation in apoptosis [41]. Caspase 3 activity in the nuclear fraction from normal rats was not statistically different from that of rats exposed to smoke and mechanical ventilation (Figure 5 and Table 1), and the content after mechanical ventilation alone was not significantly different from that of rats exposed to smoke and mechanical ventilation. An identical caspase 3 pattern was present in the cytosolic fraction (*n* = 10 per group, data not shown). Caspase 8 activity in the nuclear fraction was not different after mechanical ventilation alone or after mechanical ventilation in combination compared with controls. Cytosolic caspase 9 activity in untreated animals was not different from that of rats exposed to mechanical ventilation. The combination of the two stimuli resulted in a numerically higher caspase 9 activity level, but this difference did not reach statistical significance.

## Discussion

The results of this study indicate that the combination of cigarette smoke exposure and mechanical ventilation may interact to induce greater neutrophil influx and may increase susceptibility to acute lung injury. In particular, the combination of smoke and mechanical ventilation significantly increased airspace neutrophils compared to mechanical ventilation alone. In addition, we found less pronounced alterations in ATII cell protein expression, including significantly increased levels of DNA fragmentation. Smaller but consistent increases that did not reach statistical significance after correction for multiple comparisons were measured in the same groups for HSP70, TLR4 and caspases (Table 1).

The combination of the two stimuli triggered a significant increase in BAL neutrophils and in overall cell numbers compared to normal controls and led to increased cell differential variability between animals (Table 2). These differences were likely caused by variability introduced by both ventilation and smoke exposure, resulting in an interactive effect after combination of the two stimuli. Our findings after mechanical ventilation confirm those of a previous study in mice exposed to mechanical ventilation for 6 hours with 10 ml/kg tidal volume and PEEP of 3 cmH_2_O [42]. The results of that suggested that ventilation induced low-level BALF neutrophilia and proliferation of ATII cells, whereas ventilation did not increase apoptosis in ATII cells, as measured by terminal deoxynucleotidyl transferase deoxyuridine triphosphate nick-end labeling staining. In our previous study in rats undergoing low and high tidal volume ventilation, we found a similar small increase in BALF neutrophils in the low tidal volume ventilation group [15]. The results of our present study add to these previous studies the finding that cigarette smoke exposure further increases neutrophil recruitment following mechanical ventilation.

Compared to normal controls, the combination of smoke exposure and mechanical ventilation was associated with significant increases in BALF protein content in this 4-hour experiment. Ventilation pressures were similar between the groups (data not shown). Therefore, in this short-term experiment, differences in neutrophil inflammation did not result in measurable differences in alveolar barrier function. This observation may be due to the duration of the model, but the data do not support the hypothesis that cigarette smoke interacts with mechanical ventilation to compromise alveolar barrier function.

A novel aspect of this study is the evaluation of ATII cell protein content after the various exposures. Using MS as a discovery tool to identify potential patterns of changes in the proteome and to follow up on the results using immune techniques, we found evidence for ATII cell–specific changes in innate immune responses and regulation of pathways central to apoptosis and inflammasome function.

Following up on the MS data using immunological methods, we identified a pattern of increased levels of several markers of cell activation. However, these changes, with the exception of DNA fragments, were not statically significant after correcting for multiple comparisons. Nevertheless, the parallel increases in markers of cellular activation appeared consistent. Markers of ATII cell activation may be affected by an interaction between cigarette smoke exposure and mechanical ventilation. We identified numerical increases in TLR4 content and HSP70 content, but these differences did not reach statistical significance in our analysis. Therefore, these data are consistent with a relatively modest interaction between cigarette smoke and mechanical ventilation on alveolar epithelial cell activation. In particular, the significant increase in DNA fragments and the nonsignificant pattern of increases in caspases 3, 8 and 9 content may indicate increased apoptotic activity in the alveolar epithelium in the smoke plus ventilation group. However, the amount and variability of the changes may indicate that changes in protein content alone are not sufficient to describe the cellular response to these stimuli. Future research may need to target alterations at the posttranslational level to obtain a more comprehensive picture of the cellular response.

Previous clinical and experimental studies have identified IL-1β as an important mediator of lung injury in VILI and ARDS [43]. Our data did not show a significant increase in BALF IL-1β levels with the smoke exposure and ventilation mode selected. One potential explanation could be that the IL-1 production capacity of macrophages from alveolar lavage compared to blood macrophages is relatively limited [44] and that acute cigarette smoke exposure can induce apoptosis of alveolar macrophages [45]. In addition, our exposure levels were selected on the basis of minimal effects on our endpoints when used alone, such that interactive effects of the combined exposures could be studied. Caspases 8 and 1 have been reported as activators of IL-1β via the inflammasome pathway [41,46]. The alterations in these enzymes were not very pronounced and were not statistically significant after correction for multiple comparisons. These data suggest that inflammasome activation in ATII cells is not a major feature of the model in this study or that it does not manifest itself by changes in protein content. However, significant elevations in DNA fragments in ATII cells are consistent with an increase in alveolar epithelial cell apoptosis or injury and may reflect part of the inflammatory response driving the increase in neutrophil accumulation observed with the combination of cigarette smoke and mechanical ventilation [47-51]. The development of an oxidant/antioxidant imbalance in lung inflammation could result in cell injury and increased airspace neutrophils. An increase in catalase in BALF after cigarette smoke exposure has been described previously [37]. However, we did not find an increase in catalase in ATII cells following smoke exposure and mechanical ventilation. These data may provide some insight into the potential cellular sources of catalase during lung injury. In past studies, interactions between oxidative pathways and apoptosis have been demonstrated in an alveolar epithelial cell line [52]. Although this may be true in primary cells, our data do not support a major role for this pathway in the increased lung inflammation induced by the combination of smoke exposure and mechanical ventilation [53-55].

## Conclusions

In the samples taken from rats that had undergone a combination of cigarette smoke exposure and mechanical ventilation, BAL cell counts were significantly elevated. This finding was especially pronounced in BAL neutrophils. The increase in neutrophils in rats exposed to smoke and ventilation was present in comparisons with normal control rats and rats that had undergone mechanical ventilation alone. There were numerical increases in some markers of alveolar epithelial inflammation with the combined intervention compared with either intervention alone. However, these increases, though consistent, were largely not statistically significant after the rigorous testing for multiple comparisons that we used. This discrepancy between the increase in BAL neutrophils and cellular protein content may indicate that cellular protein content changes are relatively modest in alveolar epithelial cells. This may be because the interaction between smoke and mechanical ventilation at the alveolar epithelial cell level is small or because changes in protein content alone may not be sufficient to describe the intracellular changes. Future research may need to focus on the posttranslational level.

Cigarette smoking is a potential risk factor for ARDS [3-6] and other respiratory complications [7,8] in ICU [4,5] and perioperative settings [6-9]. Our data are consistent with the hypothesis that cigarette smoke aggravates alveolar epithelial cell injury by increasing neutrophilic inflammation in VILI. Although additional direct investigations are necessary, such an enhancing effect of prior smoke exposure on VILI could potentially contribute to the incidence of or disease course in ARDS patients who are smokers.

## Key messages

• The combination of cigarette smoke and mechanical ventilation significantly increased BAL neutrophil count and alveolar protein content as markers of lung inflammation and injury and DNR fragments as a marker of apoptosis, which indicate alveolar epithelial dysfunction.

• Our data are consistent with the hypothesis that cigarette smoke potentiates alveolar epithelial cell injury and stress responses in patients at risk for ARDS.

## Abbreviations

ARDS: Acute respiratory distress syndrome; ATII: Alveolar type II cell; BAL(F): Bronchoalveolar lavage (fluid); CSE: Cigarette smoke extract; ELISA: Enzyme-linked immune sorbent assay; FDR: False discovery rate; HSP: Heat shock protein; IL: Interleukin; iTRAQ: Isobaric tag for relative and absolute quantitation; LC: Liquid chromatography; MS: Mass spectrometry; MS-MS: Tandem mass spectrometry; NF-κB: Nuclear factor κB; NS: Nonsmoking; NV: Not ventilated; PEEP: Positive end-expiratory pressure; RP: Reverse phase; S: Smoke; TLR: Toll-like receptor; TNF: Tumor necrosis factor; TUNEL: Terminal deoxynucleotidyl transferase deoxyuridine triphosphate nick-end labeling; V: Ventilation.

## Competing interests

The authors declare that they have no competing interests, financial or otherwise.

## Authors’ contributions

All authors made substantial contributions to the conception or design of the work; the acquisition, analysis or interpretation of data; and the drafting of the manuscript or revising it critically for important intellectual content. The key areas of each author’s contributions were as follows. JH: Data acquisition, data analysis and manuscript preparation. RJC and ALB: Mass spectrometry data acquisition and analysis and manuscript preparation. TB: Data acquisition. JAF: Data analysis and manuscript preparation. All authors read and approved the final manuscript.

## Supplementary Material

Additional file 1 This picture shows the smoking apparatus and the smoking chamber. Click here for file

Additional file 2 **This table shows the mass spectrometry data.** A legend is provided at the end of the data. Click here for file

## References

[B1] Hogg JC Timens W The pathology of chronic obstructive pulmonary disease Annu Rev Pathol 2009 4 435 459 10.1146/annurev.pathol.4.110807.092145 18954287

[B2] Giovino GA The tobacco epidemic in the United States Am J Prev Med 2007 33 S318 S326 10.1016/j.amepre.2007.09.008 18021906

[B3] TenHoor T Mannino DM Moss M Risk factors for ARDS in the United States: analysis of the 1993 National Mortality Followback Study Chest 2001 119 1179 1184 10.1378/chest.119.4.1179 11296187

[B4] Iribarren C Jacobs DR Jr Sidney S Gross MD Eisner MD Cigarette smoking, alcohol consumption, and risk of ARDS: a 15-year cohort study in a managed care setting Chest 2000 117 163 168 10.1378/chest.117.1.163 10631215

[B5] Ando K Doi T Moody SY Ohkuni Y Sato S Kaneko N The effect of comorbidity on the prognosis of acute lung injury and acute respiratory distress syndrome Intern Med 2012 51 1835 1840 10.2169/internalmedicine.51.6434 22821096

[B6] Tandon S Batchelor A Bullock R Gascoigne A Griffin M Hayes N Hing J Shaw I Warnell I Baudouin SV Peri-operative risk factors for acute lung injury after elective oesophagectomy Br J Anaesth 2001 86 633 638 10.1093/bja/86.5.633 11575337

[B7] Zingg U Smithers BM Gotley DC Smith G Aly A Clough A Esterman AJ Jamieson GG Watson DI Factors associated with postoperative pulmonary morbidity after esophagectomy for cancer Ann Surg Oncol 2011 18 1460 1468 10.1245/s10434-010-1474-5 21184193

[B8] McCulloch TM Jensen NF Girod DA Tsue TT Weymuller EA Jr Risk factors for pulmonary complications in the postoperative head and neck surgery patient Head Neck 1997 19 372 377 10.1002/(SICI)1097-0347(199708)19:5<372::AID-HED2>3.0.CO;2-X 9243263

[B9] Paul DJ Jamieson GG Watson DI Devitt PG Game PA Perioperative risk analysis for acute respiratory distress syndrome after elective oesophagectomy ANZ J Surg 2011 81 700 706 10.1111/j.1445-2197.2010.05598.x 22295310

[B10] Gajic O Dabbagh O Park PK Adesanya A Chang SY Hou P Anderson H 3rd Hoth JJ Mikkelsen ME Gentile NT Gong MN Talmor D Bajwa E Watkins TR Festic E Yilmaz M Iscimen R Kaufman DA Esper AM Sadikot R Douglas I Sevransky J Malinchoc M U.S. Critical Illness and Injury Trials Group: Lung Injury Prevention Study Investigators (USCIITG-LIPS) Early identification of patients at risk of acute lung injury: evaluation of lung injury prediction score in a multicenter cohort study Am J Respir Crit Care Med 2011 183 462 470 10.1164/rccm.201004-0549OC 20802164 PMC3056224

[B11] Heckman CA Dalbey WE Pathogenesis of lesions induced in rat lung by chronic tobacco smoke inhalation J Natl Cancer Inst 1982 69 117 129 6954304 10.1093/jnci/69.1.117 PMC7204517

[B12] Wirtz HR Schmidt M Acute influence of cigarette smoke on secretion of pulmonary surfactant in rat alveolar type II cells in culture Eur Respir J 1996 9 24 32 10.1183/09031936.96.09010024 8834329

[B13] The Acute Respiratory Distress Syndrome Network Ventilation with lower tidal volumes as compared with traditional tidal volumes for acute lung injury and the acute respiratory distress syndrome N Engl J Med 2000 342 1301 1308 10793162 10.1056/NEJM200005043421801

[B14] Rocco PR Dos Santos C Pelosi P Pathophysiology of ventilator-associated lung injury Curr Opin Anaesthesiol 2012 25 123 130 10.1097/ACO.0b013e32834f8c7f 22395439

[B15] Hirsch J Hansen KC Sapru A Frank JA Chalkley RJ Fang X Trinidad JC Baker P Burlingame AL Matthay MA Impact of low and high tidal volumes on the rat alveolar epithelial type II cell proteome Am J Respir Crit Care Med 2007 175 1006 1013 10.1164/rccm.200605-621OC 17363773 PMC1899270

[B16] Hautamaki RD Kobayashi DK Senior RM Shapiro SD Requirement for macrophage elastase for cigarette smoke-induced emphysema in mice Science 1997 277 2002 2004 10.1126/science.277.5334.2002 9302297

[B17] Sulotto F Romano C Insana A Carrubba Cacciola M Cerutti A **[**Normal values of carboxyhemoglobinemia and methemoglobinemia in a sample of conscripts] [in Italian] Med Lav 1994 85 289 298 7808344

[B18] Gillis RC Weireter LJ Jr Britt RC Cole FJ Jr Collins JN Britt LD Lung protective ventilation strategies: Have we applied them in trauma patients at risk for acute lung injury and acute respiratory distress syndrome? Am Surg 2007 73 347 350 17439026

[B19] Frank JA Matthay MA Science review: mechanisms of ventilator-induced injury Crit Care 2003 7 233 241 10.1186/cc1829 12793874 PMC270664

[B20] Dobbs LG Gonzalez R Williams MC An improved method for isolating type II cells in high yield and purity Am Rev Respir Dis 1986 134 141 145 3637065 10.1164/arrd.1986.134.1.141

[B21] Morré DJ Reust T Morré DM Plasma and internal membranes from cultured mammalian cells Methods Enzymol 1994 228 448 450 7519298 10.1016/0076-6879(94)28045-2

[B22] Boylan GM Pryde JG Dobbs LG McElroy MC Identification of a novel antigen on the apical surface of rat alveolar epithelial type II and Clara cells Am J Physiol Lung Cell Mol Physiol 2001 280 L1318 L1326 11350813 10.1152/ajplung.2001.280.6.L1318

[B23] Pryde JG Phillips JH Fractionation of membrane proteins by temperature-induced phase separation in Triton X-114: application to subcellular fractions of the adrenal medulla Biochem J 1986 233 525 533 2937402 10.1042/bj2330525 PMC1153057

[B24] Watarai H Inagaki Y Kubota N Fuju K Nagafune J Yamaguchi Y Kadoya T Proteomic approach to the identification of cell membrane proteins Electrophoresis 2000 21 460 464 10.1002/(SICI)1522-2683(20000101)21:2<460::AID-ELPS460>3.0.CO;2-P 10675028

[B25] Ross PL Huang YN Marchese JN Williamson B Parker K Hattan S Khainovski N Pillai S Dey S Daniels S Purkayastha S Juhasz P Martin S Bartlet-Jones M He F Jacobson A Pappin DJ Multiplexed protein quantitation in *Saccharomyces cerevisiae* using amine-reactive isobaric tagging reagents Mol Cell Proteomics 2004 3 1154 1169 10.1074/mcp.M400129-MCP200 15385600

[B26] Jiang L He L Fountoulakis M Comparison of protein precipitation methods for sample preparation prior to proteomic analysis J Chromatogr A 2004 1023 317 320 10.1016/j.chroma.2003.10.029 14753699

[B27] Hansen KC Schmitt-Ulms G Chalkley RJ Hirsch J Baldwin MA Burlingame AL Mass spectrometric analysis of protein mixtures at low levels using cleavable ^13^C-isotope-coded affinity tag and multidimensional chromatography Mol Cell Proteomics 2003 2 299 314 12766231 10.1074/mcp.M300021-MCP200

[B28] Chalkley RJ Baker PR Hansen KC Medzihradszky KF Allen NP Rexach M Burlingame AL Comprehensive analysis of a multidimensional liquid chromatography mass spectrometry dataset acquired on a quadrupole selecting, quadrupole collision cell, time-of-flight mass spectrometer: I. How much of the data is theoretically interpretable by search engines? Mol Cell Proteomics 2005 4 1189 1193 10.1074/mcp.D500001-MCP200 15923566

[B29] Chalkley RJ Baker PR Huang L Hansen KC Allen NP Rexach M Burlingame AL Comprehensive analysis of a multidimensional liquid chromatography mass spectrometry dataset acquired on a quadrupole selecting, quadrupole collision cell, time-of-flight mass spectrometer: II. New developments in Protein Prospector allow for reliable and comprehensive automatic analysis of large datasets Mol Cell Proteomics 2005 4 1194 1204 10.1074/mcp.D500002-MCP200 15937296

[B30] UCSF Mass Spectrometry Facility. Protein Prospector Proteomics tools for mining sequence databases in conjunction with Mass Spectrometry experiments http://prospector.ucsf.edu/prospector/mshome.htm accessed April 15 2014

[B31] Benjamini Y Hochberg Y Controlling the false discovery rate: a practical and powerful approach to multiple testing J R Stat Soc Series B Stat Methodol 1995 57 289 300

[B32] Does SPSS Statistics offer multiple comparisons using the Benjamini & Hochberg method to control the false discovery rate? (script to calculate false discovery rate in SPSS). Available at http://www-01.ibm.com/support/docview.wss?uid=swg21476447 (accessed 2 April 2014)

[B33] Beere HM Wolf BB Cain K Mosser DD Mahboubi A Kuwana T Tailor P Morimoto RI Cohen GM Green DR Heat-shock protein 70 inhibits apoptosis by preventing recruitment of procaspase-9 to the Apaf-1 apoptosome Nat Cell Biol 2000 2 469 475 10.1038/35019501 10934466

[B34] Timmins JM Ozcan L Seimon TA Li G Malagelada C Backs J Backs T Bassel-Duby R Olson EN Anderson ME Tabas I Calcium/calmodulin-dependent protein kinase II links ER stress with Fas and mitochondrial apoptosis pathways J Clin Invest 2009 119 2925 2941 10.1172/JCI38857 19741297 PMC2752072

[B35] Wright JR Immunoregulatory functions of surfactant proteins Nat Rev Immunol 2005 5 58 68 10.1038/nri1528 15630429

[B36] Aoshiba K Zhou F Tsuji T Nagai A DNA damage as a molecular link in the pathogenesis of COPD in smokers Eur Respir J 2012 39 1368 1376 10.1183/09031936.00050211 22267761

[B37] Valenca SS Silva Bezerra F Lopes AA Romana-Souza B Marinho Cavalcante MC Lima AB Goncalves Koatz VL Porto LC Oxidative stress in mouse plasma and lungs induced by cigarette smoke and lipopolysaccharide Environ Res 2008 108 199 204 10.1016/j.envres.2008.07.001 18721919

[B38] Armstrong L Medford AR Uppington KM Robertson J Witherden IR Tetley TD Millar AB Expression of functional Toll-like receptor-2 and -4 on alveolar epithelial cells Am J Respir Cell Mol Biol 2004 31 241 245 10.1165/rcmb.2004-0078OC 15044215

[B39] Rahman I MacNee W Oxidative stress and regulation of glutathione in lung inflammation Eur Respir J 2000 16 534 554 10.1034/j.1399-3003.2000.016003534.x 11028671

[B40] Ramage L Guy K Expression of C-reactive protein and heat-shock protein-70 in the lung epithelial cell line A549, in response to PM10 exposure Inhal Toxicol 2004 16 447 452 10.1080/08958370490439614 15204760

[B41] Salvesen GS Caspases: opening the boxes and interpreting the arrows Cell Death Differ 2002 9 3 5 10.1038/sj.cdd.4400963 11803369

[B42] Chess PR Benson RP Maniscalco WM Wright TW O'Reilly MA Johnston CJ Murine mechanical ventilation stimulates alveolar epithelial cell proliferation Exp Lung Res 2010 36 331 341 10.3109/01902141003632332 20653468 PMC4342053

[B43] Pugin J Ricou B Steinberg KP Suter PM Martin TR Proinflammatory activity in bronchoalveolar lavage fluids from patients with ARDS, a prominent role for interleukin-1 Am J Respir Crit Care Med 1996 153 1850 1856 10.1164/ajrccm.153.6.8665045 8665045

[B44] Wewers MD Rennard SI Hance AJ Bitterman PB Crystal RG Normal human alveolar macrophages obtained by bronchoalveolar lavage have a limited capacity to release interleukin-1 J Clin Invest 1984 74 2208 2218 10.1172/JCI111647 6334697 PMC425413

[B45] Aoshiba K Tamaoki J Nagai A Acute cigarette smoke exposure induces apoptosis of alveolar macrophages Am J Physiol Lung Cell Mol Physiol 2001 281 L1392 L1401 11704535 10.1152/ajplung.2001.281.6.L1392

[B46] Dupaul-Chicoine J Saleh M A new path to IL-1β production controlled by caspase-8 Nat Immunol 2012 13 211 212 22344277 10.1038/ni.2241

[B47] Thorley AJ Ford PA Giembycz MA Goldstraw P Young A Tetley TD Differential regulation of cytokine release and leukocyte migration by lipopolysaccharide-stimulated primary human lung alveolar type II epithelial cells and macrophages J Immunol 2007 178 463 473 17182585 10.4049/jimmunol.178.1.463

[B48] Goodman RB Pugin J Lee JS Matthay MA Cytokine-mediated inflammation in acute lung injury Cytokine Growth Factor Rev 2003 14 523 535 10.1016/S1359-6101(03)00059-5 14563354

[B49] Zhou M Wan HY Huang SG Li B Li M **[**Expression of Toll-like receptor 4 in human alveolar epithelial cells and its role in cellular inflammation] [in Chinese] Zhonghua Yi Xue Za Zhi 2008 88 2112 2116 19080471

[B50] Hasday JD Bascom R Costa JJ Fitzgerald T Dubin W Bacterial endotoxin is an active component of cigarette smoke Chest 1999 115 829 835 10.1378/chest.115.3.829 10084499

[B51] Hilberath JN Carlo T Pfeffer MA Croze RH Hastrup F Levy BD Resolution of Toll-like receptor 4-mediated acute lung injury is linked to eicosanoids and suppressor of cytokine signaling 3 FASEB J 2011 25 1827 1835 10.1096/fj.10-169896 21321188 PMC3101035

[B52] Hoshino Y Mio T Nagai S Miki H Ito I Izumi T Cytotoxic effects of cigarette smoke extract on an alveolar type II cell-derived cell line Am J Physiol Lung Cell Mol Physiol 2001 281 L509 L516 11435227 10.1152/ajplung.2001.281.2.L509

[B53] Ning QM Wang XR Response of alveolar type II epithelial cells to mechanical stretch and lipopolysaccharide Respiration 2007 74 579 585 10.1159/000101724 17435381

[B54] Hammerschmidt S Kuhn H Sack U Schlenska A Gessner C Gillissen A Wirtz H Mechanical stretch alters alveolar type II cell mediator release toward a proinflammatory pattern Am J Respir Cell Mol Biol 2005 33 203 210 10.1165/rcmb.2005-0067OC 15947422

[B55] Hammerschmidt S Kuhn H Gessner C Seyfarth HJ Wirtz H Stretch-induced alveolar type II cell apoptosis: role of endogenous bradykinin and PI3K-Akt signaling Am J Respir Cell Mol Biol 2007 37 699 705 10.1165/rcmb.2006-0429OC 17630321

