# Supplementary figures and images for: Double impact of cigarette smoke and mechanical ventilation on the alveolar epithelial type II cell
Source: Crit Care. 2014 Mar 25;18(2):R50. doi: 10.1186/cc13795 (PMC4056080; doi:10.1186/cc13795)

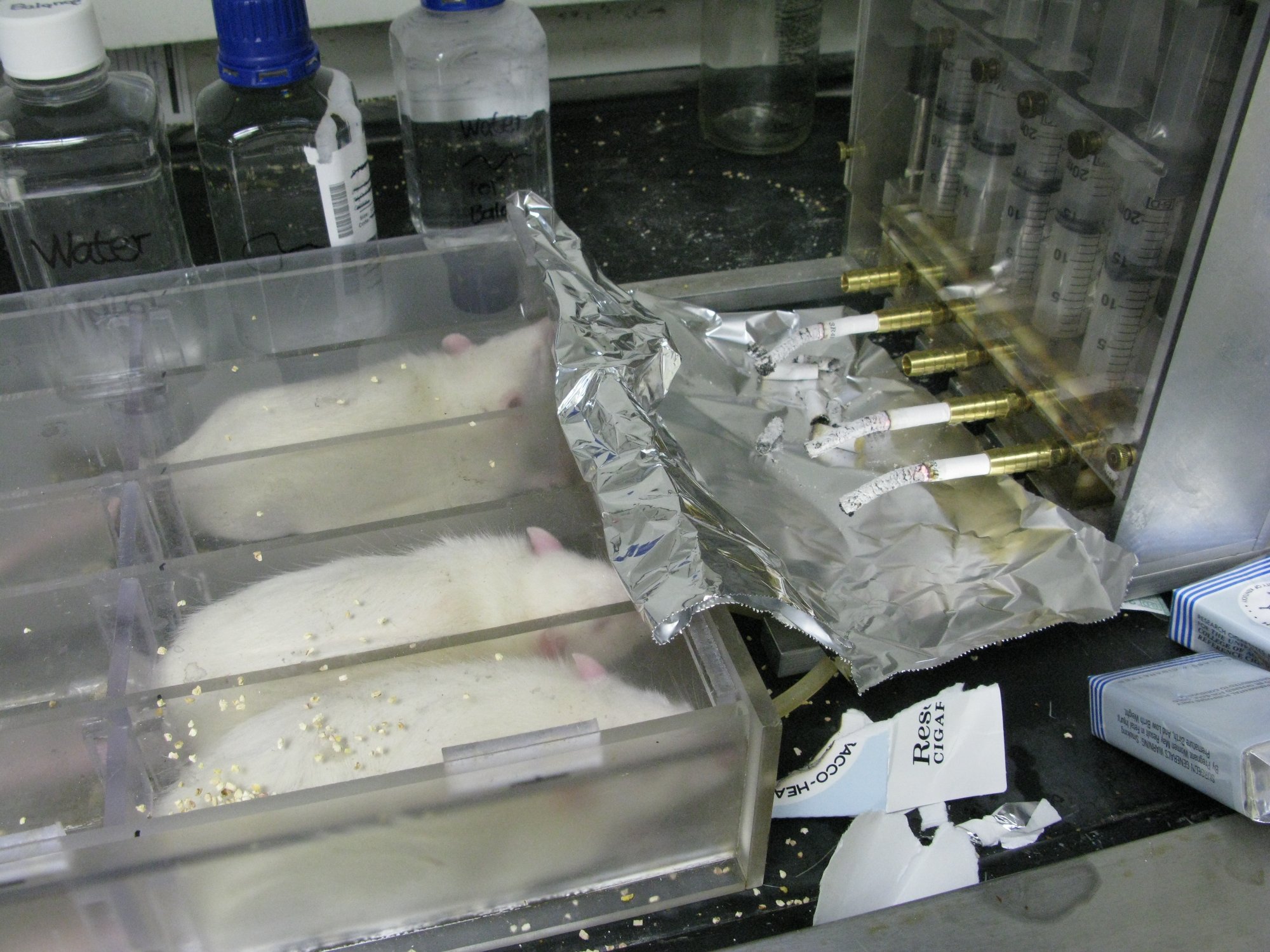

Supplement: Additional file 1 — This picture shows the smoking apparatus and the smoking chamber. [file cc13795-S1.jpeg]
