# Supplementary material for: Double impact of cigarette smoke and mechanical ventilation on the alveolar epithelial type II cell
Source: Crit Care. 2014 Mar 25;18(2):R50. doi: 10.1186/cc13795 (PMC4056080; doi:10.1186/cc13795)
Supplement: Additional file 2 — This table shows the mass spectrometry data. A legend is provided at the end of the data. [file cc13795-S2.docx]

| **Protein Name** | **Acc ession Number** | **Protein MW** | **Protein pI** | **Best Expectation Value** | **Total Peptides** | **NSNV-SNV Mean** | **NSNV-SNV STD** | **NSNV-SNV Peptides for Quantication** | **NSNV-NSV Mean** | **NSNV-NSV STD** | **NSNV-NSV Peptides for Quantication** | **NSNV-SV Mean** | **NSNV-SV STD** | **NSNV-SV Peptides for Quantication** |
| --- | --- | --- | --- | --- | --- | --- | --- | --- | --- | --- | --- | --- | --- | --- |
| **14-3-3 protein beta/alpha** | P35213 | 28055 | 4.80 | 1.9E-05 | 3 | 0.38 |  | 1 | 0.76 |  | 1 | 0.56 |  | 1 |
| **14-3-3 protein theta** | P68255 | 27779 | 4.70 | 1.9E-05 | 3 | 0.38 |  | 1 | 0.76 |  | 1 | 0.56 |  | 1 |
| **14-3-3 protein zeta/delta** | P63102 | 27771 | 4.70 | 1.9E-05 | 5 | 1.11 | 1.27 | 5 | 0.99 | 1.31 | 5 | 4.55 | 7.91 | 5 |
| **3-hydroxyacyl-CoA dehydrogenase type-2** | O70351 | 27246 | 8.90 | 1.2E-06 | 1 | 1.41 |  | 1 | 1.82 |  | 1 | 3.52 |  | 1 |
| **40S ribosomal protein S24** | P62850 | 15423 | 10.80 | 2.2E-04 | 2 | 1.64 | 0.95 | 2 | 1.71 | 1.39 | 2 | 1.91 | 1.83 | 2 |
| **40S ribosomal protein S5** | P24050 | 22879 | 9.70 | 1.2E-06 | 1 | 3.81 |  | 1 | 5.24 |  | 1 | 5.32 |  | 1 |
| **40S ribosomal protein S6** | P62755 | 28681 | 10.80 | 2.5E-09 | 1 | 0.50 |  | 1 | 4.55 |  | 1 | 1.36 |  | 1 |
| **40S ribosomal protein SA** | P38983 | 32824 | 4.80 | 1.7E-07 | 3 | 6.76 | 10.03 | 3 | 2.54 | 3.15 | 3 | 2.75 | 2.39 | 3 |
| **4-trimethylaminobutyraldehyde dehydrogenase** | Q9JLJ3 | 53653 | 6.60 | 4.7E-08 | 1 | 0.53 |  | 1 | 0.96 |  | 1 | 0.51 |  | 1 |
| **60S ribosomal protein L19** | P84100 | 23466 | 11.50 | 4.2E-05 | 1 | 2.84 |  | 1 | 2.39 |  | 1 | 3.75 |  | 1 |
| **78 kDa glucose-regulated protein** | P06761 | 72348 | 5.10 | 1.3E-06 | 6 | 22.02 | 43.56 | 6 | 16.94 | 32.86 | 6 | 17.85 | 30.25 | 6 |
| **Aconitate hydratase, mitochondrial** | Q9ER34 | 85434 | 7.90 | 1.5E-05 | 1 | 0.33 |  | 1 | 0.44 |  | 1 | 0.32 |  | 1 |
| **Actin, cytoplasmic 1** | P60711 | 41737 | 5.30 | 1.0E-09 | 16 | 2.83 | 4.06 | 14 | 2.23 | 3.05 | 14 | 5.95 | 9.29 | 16 |
| **Adenine phosphoribosyltransferase** | P36972 | 19546 | 6.20 | 2.5E-03 | 1 | 15.05 |  | 1 | 22.22 |  | 1 | 67.22 |  | 1 |
| **Adenylate kinase 2, mitochondrial** | P29410 | 26380 | 6.30 | 2.5E-09 | 1 | 0.23 |  | 1 | 0.76 |  | 1 | 1.03 |  | 1 |
| **Adenylyl cyclase-associated protein 1** | Q08163 | 51589 | 7.20 | 2.5E-09 | 1 | 35.23 |  | 1 | 61.76 |  | 1 | 256.60 |  | 1 |
| **ADP/ATP translocase 1** | Q05962 | 32990 | 9.80 | 3.7E-06 | 2 | 1.51 | 0.27 | 2 | 1.37 | 0.12 | 2 | 4.93 | 4.09 | 2 |
| **ADP-ribosylation factor 3** | P61206 | 20601 | 6.80 | 3.7E-06 | 1 | 0.43 |  | 1 | 4.20 |  | 1 | 1.08 |  | 1 |
| **Alcohol dehydrogenase class-3** | P12711 | 39576 | 7.50 | 7.5E-07 | 1 |  |  | 0 |  |  | 0 |  |  | 0 |
| **Aldehyde dehydrogenase, cytosolic 1** | P13601 | 54560 | 7.10 | 1.3E-06 | 4 | 0.45 | 0.19 | 3 | 0.34 | 0.15 | 3 | 0.90 | 0.45 | 3 |
| **Aldehyde dehydrogenase, mitochondrial** | P11884 | 56489 | 6.60 | 1.3E-06 | 7 | 2.04 | 2.17 | 7 | 1.50 | 1.62 | 7 | 1.90 | 1.40 | 7 |
| **Alpha-actinin-4** | Q9QXQ0 | 104916 | 5.30 | 1.2E-06 | 1 | 0.32 |  | 1 | 0.64 |  | 1 | 0.35 |  | 1 |
| **Ancient ubiquitous protein 1** | A1L134 | 46200 | 8.70 | 2.2E-04 | 1 | 6.61 |  | 1 | 6.51 |  | 1 | 5.70 |  | 1 |
| **Annexin A1** | P07150 | 38830 | 7.00 | 1.0E-09 | 8 | 1.84 | 1.70 | 8 | 1.48 | 1.21 | 8 | 2.20 | 1.84 | 8 |
| **Annexin A2** | Q07936 | 38679 | 7.50 | 8.6E-05 | 4 | 1.36 | 0.56 | 3 | 0.99 | 0.37 | 3 | 1.43 | 1.26 | 3 |
| **Annexin A4** | P55260 | 35849 | 5.30 | 2.2E-04 | 1 | 0.29 |  | 1 | 0.60 |  | 1 | 0.44 |  | 1 |
| **Annexin A5** | P14668 | 35745 | 4.90 | 1.3E-06 | 7 | 0.76 | 1.09 | 6 | 0.62 | 0.81 | 6 | 2.04 | 2.91 | 7 |
| **Annexin A6** | P48037 | 75755 | 5.40 | 7.5E-07 | 1 |  |  | 0 |  |  | 0 |  |  | 0 |
| **Aspartate aminotransferase, mitochondrial** | P00507 | 47315 | 9.10 | 3.7E-06 | 4 | 0.81 |  | 1 | 1.84 |  | 1 | 2.59 |  | 1 |
| **ATP synthase subunit alpha, mitochondrial** | P15999 | 59754 | 9.20 | 8.6E-05 | 4 | 2.94 | 1.35 | 4 | 1.91 | 0.50 | 4 | 2.84 | 1.59 | 4 |
| **ATP synthase subunit b, mitochondrial** | P19511 | 28869 | 9.40 | 3.7E-06 | 2 | 5.71 |  | 1 | 4.74 |  | 1 | 15.91 |  | 1 |
| **ATP synthase subunit beta, mitochondrial** | P10719 | 56354 | 5.20 | 8.6E-05 | 2 | 13.48 | 16.20 | 2 | 5.10 | 5.04 | 2 | 22.44 | 30.99 | 2 |
| **ATP-dependent RNA helicase DDX39A** | Q5U216 | 49110 | 5.50 | 2.5E-09 | 2 | 0.71 |  | 1 | 11.81 |  | 1 | 1.92 |  | 1 |
| **Beta-glucuronidase** | P06760 | 74794 | 6.30 | 1.3E-07 | 1 |  |  | 0 |  |  | 0 |  |  | 0 |
| **Calmodulin** | P62161 | 16838 | 4.10 | 1.9E-05 | 5 | 5.57 | 6.86 | 5 | 3.24 | 5.17 | 5 | 15.30 | 25.09 | 4 |
| **Calreticulin** | P18418 | 47996 | 4.30 | 1.7E-07 | 5 | 1.52 | 1.35 | 5 | 1.22 | 0.97 | 5 | 2.84 | 3.10 | 5 |
| **Carbonic anhydrase 2** | P27139 | 29114 | 6.90 | 5.5E-08 | 1 | 3.08 |  | 1 | 0.90 |  | 1 | 1.51 |  | 1 |
| **Carbonyl reductase [NADPH] 1** | P47727 | 30578 | 8.20 | 1.5E-05 | 1 | 4.96 |  | 1 | 3.29 |  | 1 | 5.30 |  | 1 |
| **Carboxylesterase 3** | P16303 | 62148 | 6.10 | 1.3E-06 | 6 | 2.30 | 2.87 | 5 | 2.09 | 2.04 | 5 | 7.47 | 11.55 | 6 |
| **Catalase** | P04762 | 59758 | 7.10 | 1.0E-09 | 8 | 3.11 | 5.24 | 8 | 2.45 | 3.94 | 8 | 2.68 | 3.23 | 8 |
| **Cathepsin Z** | Q9R1T3 | 34195 | 6.70 | 2.2E-04 | 1 | 0.55 |  | 1 | 2.24 |  | 1 | 0.44 |  | 1 |
| **Cationic trypsin-3** | P08426 | 26269 | 7.50 | 7.5E-07 | 1 |  |  | 0 |  |  | 0 |  |  | 0 |
| **Chromodomain-helicase-DNA-binding protein 8** | Q9JIX5 | 290695 | 6.00 | 3.8E-07 | 1 | 0.04 |  | 1 | 0.06 |  | 1 | 0.02 |  | 1 |
| **Citrate synthase, mitochondrial** | Q8VHF5 | 51867 | 8.50 | 2.2E-04 | 2 | 1.28 | 1.33 | 2 | 1.12 | 0.80 | 2 | 0.72 | 0.34 | 2 |
| **Cofilin-1** | P45592 | 18533 | 8.20 | 2.2E-04 | 2 | 1.65 | 1.23 | 2 | 1.01 | 1.27 | 2 | 8.73 | 5.94 | 2 |
| **Core histone macro-H2A.1** | Q02874 | 39504 | 9.80 | 1.5E-05 | 1 | 0.62 |  | 1 | 1.32 |  | 1 | 0.80 |  | 1 |
| **Cytochrome c oxidase subunit 4 isoform 1, mitochondrial** | P10888 | 19515 | 9.40 | 4.2E-05 | 1 |  |  | 0 |  |  | 0 |  |  | 0 |
| **Cytochrome c oxidase subunit 5A, mitochondrial** | P11240 | 16130 | 6.10 | 4.7E-08 | 2 | 1.22 |  | 1 | 1.39 |  | 1 | 6.20 |  | 1 |
| **Desmin** | P48675 | 53457 | 5.20 | 1.0E-09 | 3 | 7.30 | 10.23 | 3 | 7.14 | 6.71 | 3 | 6.12 | 7.65 | 3 |
| **Destrin** | Q7M0E3 | 18534 | 8.20 | 2.1E-05 | 1 | 0.31 |  | 1 | 1.26 |  | 1 | 0.39 |  | 1 |
| **Dihydrolipoyllysine-residue acetyltransferase component of pyruvate dehydrogenase complex, mitochondrial** | P08461 | 67166 | 8.80 | 1.2E-06 | 1 | 1.04 |  | 1 | 1.80 |  | 1 | 2.90 |  | 1 |
| **Dihydrolipoyllysine-residue succinyltransferase component of 2-oxoglutarate dehydrogenase complex, mitochondrial** | Q01205 | 48926 | 8.90 | 1.5E-05 | 2 |  |  | 0 |  |  | 0 |  |  | 0 |
| **Dipeptidyl peptidase 1** | P80067 | 52236 | 6.40 | 2.5E-09 | 1 | 0.53 |  | 1 | 0.73 |  | 1 | 0.55 |  | 1 |
| **Dolichyl-diphosphooligosaccharide--protein glycosyltransferase subunit 1** | P07153 | 68305 | 6.10 | 2.2E-04 | 2 | 19.41 |  | 1 | 4.70 |  | 1 | 14.05 |  | 1 |
| **Dolichyl-diphosphooligosaccharide--protein glycosyltransferase subunit DAD1** | P61805 | 12497 | 6.50 | 2.5E-09 | 1 | 5.90 |  | 1 | 7.00 |  | 1 | 31.09 |  | 1 |
| **Echinoderm microtubule-associated protein-like 2** | Q6P6T4 | 70711 | 5.90 | 1.2E-06 | 1 | 0.34 |  | 1 | 0.69 |  | 1 | 2.45 |  | 1 |
| **Electron transfer flavoprotein subunit beta** | Q68FU3 | 27688 | 7.60 | 4.0E-07 | 1 |  |  | 0 |  |  | 0 |  |  | 0 |
| **Elongation factor 1-alpha 1** | P62630 | 50114 | 9.10 | 1.3E-06 | 5 | 6.29 | 7.45 | 5 | 5.09 | 5.46 | 5 | 3.49 | 2.33 | 5 |
| **Elongation factor 2** | P05197 | 95285 | 6.40 | 1.7E-07 | 4 | 1.40 | 1.19 | 4 | 8.40 | 15.14 | 4 | 4.21 | 4.24 | 4 |
| **Endoplasmic reticulum resident protein 29** | P52555 | 28575 | 6.20 | 4.0E-07 | 1 |  |  | 0 |  |  | 0 |  |  | 0 |
| **Endoplasmin** | Q66HD0 | 92772 | 4.70 | 1.3E-06 | 6 | 1.59 | 1.89 | 6 | 0.70 | 0.52 | 6 | 1.18 | 1.12 | 6 |
| **Enoyl-CoA delta isomerase 1, mitochondrial** | P23965 | 32255 | 9.60 | 4.7E-08 | 1 | 0.23 |  | 1 | 0.55 |  | 1 | 0.41 |  | 1 |
| **Epoxide hydrolase 1** | P07687 | 52582 | 8.60 | 2.5E-09 | 1 | 1.67 |  | 1 | 2.46 |  | 1 | 10.19 |  | 1 |
| **Ezrin** | P31977 | 69391 | 5.80 | 5.5E-08 | 2 | 1.97 | 2.50 | 2 | 1.65 | 1.66 | 2 | 5.37 | 6.85 | 2 |
| **Fatty acid synthase** | P12785 | 272652 | 6.00 | 1.7E-07 | 7 | 1.40 | 1.41 | 7 | 1.17 | 1.02 | 7 | 1.90 | 1.36 | 7 |
| **Fatty acid-binding protein, epidermal** | P55053 | 15060 | 6.70 | 3.7E-06 | 1 | 1.27 |  | 1 | 10.09 |  | 1 | 3.17 |  | 1 |
| **Ferritin heavy chain** | P19132 | 21127 | 5.90 | 4.0E-07 | 1 |  |  | 0 |  |  | 0 |  |  | 0 |
| **Fructose-bisphosphate aldolase A** | P05065 | 39352 | 8.30 | 8.6E-05 | 3 | 0.37 | 0.15 | 2 | 12.17 | 20.59 | 3 | 8.65 | 11.16 | 3 |
| **Glial fibrillary acidic protein** | P47819 | 49958 | 5.40 | 4.0E-07 | 1 |  |  | 0 |  |  | 0 |  |  | 0 |
| **Glucose-6-phosphate isomerase** | Q6P6V0 | 62828 | 7.40 | 3.7E-06 | 1 | 0.43 |  | 1 | 5.56 |  | 1 | 2.48 |  | 1 |
| **Glutathione S-transferase alpha-3** | P04904 | 25320 | 8.80 | 8.6E-05 | 7 | 2.42 | 3.34 | 3 | 2.77 | 4.16 | 3 | 2.04 | 2.57 | 3 |
| **Glutathione S-transferase Mu 2** | P08010 | 25703 | 6.90 | 6.3E-04 | 4 | 4.25 | 4.91 | 4 | 3.24 | 3.76 | 4 | 3.16 | 3.72 | 4 |
| **GTP:AMP phosphotransferase, mitochondrial** | P29411 | 25439 | 8.90 | 5.5E-08 | 1 | 0.50 |  | 1 | 0.51 |  | 1 | 0.30 |  | 1 |
| **GTP-binding nuclear protein Ran** | P62828 | 24423 | 7.00 | 3.7E-06 | 1 | 0.79 |  | 1 | 1.78 |  | 1 | 2.19 |  | 1 |
| **Heat shock 70 kDa protein 1A/1B** | Q07439 | 70186 | 5.60 | 1.3E-06 | 3 | 6.68 | 7.27 | 3 | 8.18 | 4.48 | 3 | 7.95 | 6.91 | 3 |
| **Heat shock 70 kDa protein 1-like** | P55063 | 70550 | 5.90 | 1.3E-06 | 3 | 6.86 | 7.18 | 3 | 8.32 | 4.24 | 3 | 8.11 | 6.75 | 3 |
| **Heat shock cognate 71 kDa protein** | P63018 | 70872 | 5.40 | 1.3E-06 | 4 | 4.14 | 7.18 | 4 | 3.24 | 5.37 | 4 | 5.43 | 7.24 | 4 |
| **Heat shock protein 60 kDa , mitochondrial** | P63039 | 60956 | 5.90 | 1.7E-07 | 4 | 1.97 | 2.46 | 4 | 1.59 | 1.78 | 4 | 4.53 | 6.50 | 4 |
| **Heat shock protein beta-1** | P42930 | 22893 | 6.10 | 8.6E-05 | 3 | 1.59 | 0.78 | 3 | 1.76 | 0.99 | 3 | 2.60 | 0.41 | 3 |
| **Heat shock protein HSP 90-alpha** | P82995 | 84816 | 4.90 | 1.3E-06 | 5 | 1.12 | 0.94 | 5 | 2.60 | 3.85 | 5 | 2.12 | 0.95 | 5 |
| **Heat shock protein HSP 90-beta** | P34058 | 83282 | 5.00 | 1.0E-09 | 6 | 1.78 | 1.88 | 6 | 2.80 | 3.50 | 6 | 2.11 | 0.98 | 6 |
| **Heat shock protein10 kDa, mitochondrial** | P26772 | 10902 | 8.90 | 2.2E-04 | 2 | 0.90 |  | 1 | 1.03 |  | 1 | 0.91 |  | 1 |
| **Heat shock-related 70 kDa protein 2** | P14659 | 69642 | 5.50 | 1.3E-06 | 3 | 5.43 | 8.21 | 3 | 4.24 | 6.10 | 3 | 7.03 | 7.96 | 3 |
| **Hemoglobin subunit alpha-1/2** | P01946 | 15329 | 7.80 | 1.0E-09 | 7 | 1.59 | 0.87 | 5 | 1.27 | 0.57 | 5 | 1.99 | 1.69 | 7 |
| **Hemoglobin subunit beta-1** | P02091 | 15980 | 7.90 | 1.0E-09 | 6 | 1.78 | 1.51 | 4 | 8.59 | 15.11 | 4 | 6.43 | 8.87 | 5 |
| **Hemoglobin subunit beta-2** | P11517 | 15983 | 8.90 | 1.0E-09 | 5 | 6.70 | 10.70 | 4 | 1.05 | 1.20 | 3 | 2.36 | 1.43 | 5 |
| **Heterogeneous nuclear ribonucleoprotein A3** | Q6URK4 | 39652 | 9.10 | 2.2E-04 | 1 | 0.40 |  | 1 | 0.64 |  | 1 | 0.37 |  | 1 |
| **Heterogeneous nuclear ribonucleoprotein D0** | Q9JJ54 | 38192 | 7.60 | 1.2E-06 | 2 | 1.20 |  | 1 | 8.86 |  | 1 | 2.83 |  | 1 |
| **Heterogeneous nuclear ribonucleoprotein F** | Q794E4 | 45730 | 5.30 | 2.1E-05 | 1 | 0.23 |  | 1 | 0.73 |  | 1 | 0.58 |  | 1 |
| **Heterogeneous nuclear ribonucleoprotein H2** | Q6AY09 | 49294 | 5.90 | 8.6E-05 | 2 | 10.69 | 14.75 | 2 | 8.37 | 10.79 | 2 | 10.58 | 14.37 | 2 |
| **Heterogeneous nuclear ribonucleoprotein K** | P61980 | 50977 | 5.40 | 1.7E-07 | 3 | 0.91 | 0.63 | 3 | 1.50 | 1.47 | 3 | 1.29 | 0.77 | 3 |
| **Heterogeneous nuclear ribonucleoproteins A2/B1** | A7VJC2 | 37478 | 9.00 | 1.3E-06 | 7 | 7.84 | 17.38 | 7 | 6.00 | 13.13 | 7 | 6.92 | 15.76 | 7 |
| **High mobility group protein B2** | P52925 | 24159 | 6.90 | 1.3E-07 | 1 |  |  | 0 |  |  | 0 |  |  | 0 |
| **Histidine triad nucleotide-binding protein 1** | P62959 | 13777 | 6.40 | 6.2E-05 | 1 |  |  | 0 |  |  | 0 |  |  | 0 |
| **Histone H1.0** | P43278 | 20885 | 10.90 | 2.1E-05 | 1 | 0.18 |  | 1 | 0.58 |  | 1 | 0.28 |  | 1 |
| **Histone H1.2** | P15865 | 21988 | 11.10 | 1.0E-09 | 5 | 14.17 | 11.79 | 3 | 8.61 | 5.32 | 3 | 5.67 | 4.54 | 3 |
| **Histone H2A.J** | A9UMV8 | 14046 | 11.00 | 1.7E-07 | 4 | 3.22 | 3.37 | 4 | 2.58 | 2.42 | 4 | 1.44 | 1.01 | 4 |
| **Histone H2B type 1** | Q00715 | 13990 | 10.40 | 1.9E-05 | 6 | 3.54 | 3.44 | 5 | 2.51 | 2.64 | 5 | 17.10 | 31.62 | 6 |
| **Histone H3.3** | P84245 | 15328 | 11.30 | 2.1E-05 | 5 | 65.99 |  | 1 | 2.52 |  | 1 | 11.02 |  | 1 |
| **Histone H4** | P62804 | 11367 | 11.40 | 6.3E-04 | 4 | 1.24 | 0.78 | 4 | 2.06 | 2.32 | 4 | 1.43 | 1.00 | 4 |
| **Hydroxyacyl-coenzyme A dehydrogenase, mitochondrial** | Q9WVK7 | 34448 | 8.80 | 2.1E-05 | 2 | 3.02 | 3.90 | 2 | 2.43 | 2.75 | 2 | 2.79 | 3.36 | 2 |
| **Hypoxia up-regulated protein 1** | Q63617 | 111290 | 5.10 | 5.5E-08 | 1 | 0.48 |  | 1 | 0.82 |  | 1 | 0.72 |  | 1 |
| **Isocitrate dehydrogenase [NADP], mitochondrial** | P56574 | 50968 | 8.90 | 1.9E-05 | 5 | 2.06 | 1.54 | 5 | 1.65 | 1.04 | 5 | 2.51 | 2.14 | 5 |
| **Isovaleryl-CoA dehydrogenase, mitochondrial** | P12007 | 46436 | 8.00 | 2.2E-04 | 1 | 0.35 |  | 1 | 0.52 |  | 1 | 0.33 |  | 1 |
| **Keratin, type I cytoskeletal 18** | Q5BJY9 | 47762 | 5.20 | 1.0E-09 | 12 | 1.66 | 1.50 | 12 | 1.29 | 1.16 | 12 | 3.43 | 3.39 | 12 |
| **Keratin, type I cytoskeletal 19** | Q63279 | 44636 | 5.20 | 1.0E-09 | 13 | 2.12 | 2.65 | 13 | 1.62 | 2.00 | 13 | 3.56 | 4.06 | 13 |
| **Keratin, type II cytoskeletal 8** | Q10758 | 54019 | 5.80 | 1.0E-09 | 13 | 3.24 | 5.31 | 12 | 2.47 | 4.01 | 12 | 4.44 | 5.13 | 12 |
| **Lamin-B receptor** | O08984 | 70725 | 9.60 | 5.5E-08 | 1 | 1.23 |  | 1 | 0.81 |  | 1 | 3.78 |  | 1 |
| **Lamin-B1** | P70615 | 66607 | 5.20 | 1.0E-09 | 5 | 0.80 | 0.22 | 3 | 1.10 | 0.74 | 3 | 1.92 | 1.15 | 3 |
| **Leucine zipper putative tumor suppressor 2** | Q3LUD4 | 72494 | 6.40 | 1.3E-07 | 1 |  |  | 0 |  |  | 0 |  |  | 0 |
| **Leukotriene A-4 hydrolase** | P30349 | 69177 | 5.70 | 4.0E-07 | 1 |  |  | 0 |  |  | 0 |  |  | 0 |
| **L-lactate dehydrogenase A chain** | P04642 | 36451 | 8.40 | 1.7E-07 | 3 | 0.97 | 0.04 | 2 | 1.82 | 1.56 | 2 | 1.21 | 1.19 | 2 |
| **Lysozyme C-1** | P00697 | 16729 | 9.30 | 4.7E-08 | 2 | 0.50 |  | 1 | 2.33 |  | 1 | 1.54 |  | 1 |
| **Malate dehydrogenase, cytoplasmic** | O88989 | 36483 | 6.20 | 1.2E-06 | 1 | 1.02 |  | 1 | 0.42 |  | 1 | 2.62 |  | 1 |
| **Malate dehydrogenase, mitochondrial** | P04636 | 35684 | 8.90 | 2.1E-05 | 3 | 1.86 | 2.09 | 2 | 1.53 | 1.40 | 2 | 0.73 | 0.46 | 2 |
| **Mast cell protease 2** | P00770 | 27102 | 8.20 | 2.5E-09 | 1 | 0.34 |  | 1 | 4.83 |  | 1 | 1.59 |  | 1 |
| **Mitochondrial-processing peptidase subunit beta** | Q03346 | 54266 | 6.30 | 6.2E-05 | 1 |  |  | 0 |  |  | 0 |  |  | 0 |
| **Moesin** | O35763 | 67740 | 6.20 | 1.7E-07 | 4 | 1.42 | 1.46 | 4 | 2.15 | 2.08 | 4 | 3.28 | 1.45 | 4 |
| **Myosin light chain 3** | P16409 | 22156 | 5.00 | 1.7E-04 | 1 |  |  | 0 |  |  | 0 |  |  | 0 |
| **Myosin regulatory light chain RLC-A** | P13832 | 19896 | 4.70 | 3.8E-07 | 1 | 0.26 |  | 1 | 0.79 |  | 1 | 0.42 |  | 1 |
| **Myosin-10** | Q9JLT0 | 228967 | 5.50 | 6.2E-05 | 1 |  |  | 0 |  |  | 0 |  |  | 0 |
| **Myosin-9** | Q62812 | 226340 | 5.50 | 2.1E-05 | 4 | 0.65 | 0.43 | 3 | 0.74 | 0.22 | 3 | 2.43 | 1.89 | 3 |
| **N(G),N(G)-dimethylarginine dimethylaminohydrolase 2** | Q6MG60 | 29688 | 5.70 | 1.7E-04 | 1 |  |  | 0 |  |  | 0 |  |  | 0 |
| **Na(+)/H(+) exchange regulatory cofactor NHE-RF1** | Q9JJ19 | 38831 | 5.70 | 2.2E-04 | 1 | 33.51 |  | 1 | 8.17 |  | 1 | 9.62 |  | 1 |
| **NAD(P)H dehydrogenase [quinone] 1** | P05982 | 30947 | 8.40 | 2.5E-03 | 1 | 1.25 |  | 1 | 1.16 |  | 1 | 1.55 |  | 1 |
| **Nucleophosmin** | P13084 | 32560 | 4.60 | 4.7E-08 | 1 | 1.37 |  | 1 | 15.37 |  | 1 | 6.33 |  | 1 |
| **Peptidyl-prolyl cis-trans isomerase A** | P10111 | 17875 | 8.30 | 2.2E-04 | 2 | 1.42 |  | 1 | 2.37 |  | 1 | 2.63 |  | 1 |
| **Peptidyl-prolyl cis-trans isomerase B** | P24368 | 23803 | 9.50 | 2.1E-05 | 2 | 3.93 | 4.17 | 2 | 4.02 | 4.63 | 2 | 8.03 | 3.52 | 2 |
| **Peroxiredoxin-1** | Q63716 | 22110 | 8.30 | 1.9E-05 | 6 | 0.85 | 0.95 | 5 | 12.30 | 28.55 | 6 | 11.85 | 17.28 | 6 |
| **Peroxiredoxin-2** | P35704 | 21784 | 5.30 | 8.6E-05 | 3 | 1.23 | 1.61 | 3 | 1.02 | 1.15 | 3 | 1.11 | 0.90 | 3 |
| **Peroxiredoxin-6** | O35244 | 24819 | 5.60 | 1.9E-05 | 6 | 1.15 | 0.93 | 6 | 0.92 | 0.66 | 6 | 1.13 | 0.56 | 6 |
| **Phosphate carrier protein, mitochondrial** | P16036 | 39446 | 9.40 | 2.5E-03 | 1 | 1.71 |  | 1 | 2.74 |  | 1 | 7.66 |  | 1 |
| **Phosphoglycerate kinase 1** | P16617 | 44539 | 8.00 | 1.7E-04 | 1 |  |  | 0 |  |  | 0 |  |  | 0 |
| **Polyadenylate-binding protein 1** | Q9EPH8 | 70702 | 9.50 | 5.5E-08 | 1 | 1.35 |  | 1 | 15.76 |  | 1 | 4.33 |  | 1 |
| **Polyubiquitin-B** | P0CG51 | 34369 | 6.90 | 8.6E-05 | 3 | 4.36 | 3.20 | 3 | 3.92 | 1.91 | 3 | 2.79 | 1.26 | 3 |
| **PRA1 family protein 3** | Q9ES40 | 21549 | 9.60 | 2.5E-03 | 1 |  |  | 0 |  |  | 0 |  |  | 0 |
| **Prelamin-A/C** | P48679 | 74324 | 6.50 | 1.0E-09 | 13 | 3.00 | 3.32 | 13 | 2.30 | 2.48 | 13 | 4.44 | 5.13 | 13 |
| **Profilin-1** | P62963 | 14957 | 8.50 | 7.5E-07 | 1 |  |  | 0 |  |  | 0 |  |  | 0 |
| **Prohibitin** | P67779 | 29820 | 5.60 | 2.1E-05 | 3 | 27.14 | 36.51 | 2 | 10.22 | 13.03 | 2 | 8.19 | 6.66 | 2 |
| **Prohibitin-2** | Q5XIH7 | 33313 | 9.80 | 3.8E-07 | 1 | 1.01 |  | 1 | 2.13 |  | 1 | 4.46 |  | 1 |
| **Prolactin-6A1** | P24800 | 27148 | 7.10 | 1.5E-05 | 1 | 0.30 |  | 1 | 1.48 |  | 1 | 2.10 |  | 1 |
| **Proteasome subunit alpha type-4** | P21670 | 29498 | 7.60 | 4.2E-05 | 1 |  |  | 0 |  |  | 0 |  |  | 0 |
| **Proteasome subunit beta type-1** | P18421 | 26480 | 6.90 | 5.5E-08 | 1 | 0.82 |  | 1 | 8.45 |  | 1 | 3.46 |  | 1 |
| **Proteasome subunit beta type-4** | P34067 | 29198 | 6.40 | 2.2E-04 | 1 | 34.91 |  | 1 | 58.14 |  | 1 | 89.09 |  | 1 |
| **Protein disulfide-isomerase** | P04785 | 56952 | 4.80 | 1.7E-07 | 4 | 2.56 | 1.76 | 4 | 2.01 | 1.20 | 4 | 3.66 | 2.80 | 4 |
| **Protein disulfide-isomerase A3** | P11598 | 56624 | 5.90 | 1.0E-09 | 11 | 2.58 | 2.78 | 10 | 1.95 | 2.11 | 10 | 4.52 | 4.50 | 10 |
| **Protein disulfide-isomerase A6** | Q63081 | 48174 | 5.00 | 1.2E-06 | 2 |  |  | 0 |  |  | 0 |  |  | 0 |
| **Protein mago nashi homolog** | Q27W02 | 17164 | 5.70 | 3.8E-07 | 1 | 6.50 |  | 1 | 6.46 |  | 1 | 5.41 |  | 1 |
| **Protein S100-A11** | Q6B345 | 11065 | 5.60 | 3.8E-07 | 2 | 0.23 |  | 1 | 0.70 |  | 1 | 0.48 |  | 1 |
| **Protein S100-A6** | P05964 | 10035 | 5.30 | 1.7E-07 | 5 | 13.06 | 25.24 | 5 | 10.76 | 18.71 | 5 | 45.40 | 95.24 | 5 |
| **Protein S100-A9** | P50116 | 13145 | 7.10 | 1.7E-04 | 1 |  |  | 0 |  |  | 0 |  |  | 0 |
| **Proteolipid protein 2** | Q6P742 | 16557 | 6.70 | 5.5E-08 | 1 | 1.64 |  | 1 | 3.92 |  | 1 | 5.25 |  | 1 |
| **Prothymosin alpha** | P06302 | 12382 | 3.80 | 2.1E-05 | 1 | 3.35 |  | 1 | 2.21 |  | 1 | 6.93 |  | 1 |
| **Pulmonary surfactant-associated protein A** | P08427 | 26289 | 4.80 | 7.5E-07 | 1 |  |  | 0 |  |  | 0 |  |  | 0 |
| **Pulmonary surfactant-associated protein B** | P22355 | 41590 | 6.20 | 8.6E-05 | 2 | 9.49 | 12.61 | 2 | 7.72 | 8.79 | 2 | 14.74 | 18.57 | 2 |
| **Pulmonary surfactant-associated protein D** | P35248 | 37561 | 6.80 | 1.2E-06 | 1 | 27.20 |  | 1 | 6.91 |  | 1 | 10.91 |  | 1 |
| **Purine nucleoside phosphorylase** | P85973 | 32302 | 6.50 | 8.6E-05 | 2 | 7.27 | 9.87 | 2 | 5.20 | 7.03 | 2 | 4.75 | 6.08 | 2 |
| **Pyruvate carboxylase, mitochondrial** | P52873 | 129778 | 6.30 | 1.7E-04 | 1 |  |  | 0 |  |  | 0 |  |  | 0 |
| **Pyruvate kinase isozymes M1/M2** | P11980 | 57818 | 6.60 | 8.6E-05 | 3 | 0.57 | 0.26 | 3 | 1.83 | 2.28 | 3 | 2.08 | 1.19 | 3 |
| **Rab GDP dissociation inhibitor alpha** | P50398 | 50537 | 5.00 | 2.5E-09 | 1 | 0.33 |  | 1 | 0.80 |  | 1 | 0.55 |  | 1 |
| **Ras-related C3 botulinum toxin substrate 1** | Q6RUV5 | 21450 | 8.80 | 1.5E-05 | 1 | 0.33 |  | 1 | 0.91 |  | 1 | 0.78 |  | 1 |
| **Ras-related protein Rab-6A** | Q9WVB1 | 23590 | 5.40 | 3.7E-06 | 1 | 2.90 |  | 1 | 1.54 |  | 1 | 2.03 |  | 1 |
| **Regulator of G-protein signaling 7-binding protein** | Q5FVH8 | 28966 | 8.80 | 4.2E-05 | 1 |  |  | 0 |  |  | 0 |  |  | 0 |
| **Retinal dehydrogenase 1** | P51647 | 54459 | 7.90 | 1.9E-05 | 4 | 0.45 | 0.19 | 3 | 0.34 | 0.15 | 3 | 0.90 | 0.45 | 3 |
| **SEC14-like protein 3** | Q9Z1J8 | 46028 | 5.60 | 6.2E-05 | 1 |  |  | 0 |  |  | 0 |  |  | 0 |
| **Serine/arginine-rich splicing factor 5** | Q09167 | 30892 | 11.60 | 4.0E-07 | 1 |  |  | 0 |  |  | 0 |  |  | 0 |
| **Serotransferrin** | P12346 | 76396 | 7.10 | 6.2E-05 | 1 |  |  | 0 |  |  | 0 |  |  | 0 |
| **Sodium/potassium-transporting ATPase subunit alpha-1** | P06685 | 113055 | 5.30 | 1.2E-06 | 1 | 7.39 |  | 1 | 8.79 |  | 1 | 28.62 |  | 1 |
| **Solute carrier family 13 member 1** | Q07782 | 66060 | 7.10 | 1.7E-04 | 1 |  |  | 0 |  |  | 0 |  |  | 0 |
| **Splicing factor 3B subunit 4** | Q6AYL5 | 44356 | 8.50 | 4.2E-05 | 1 |  |  | 0 |  |  | 0 |  |  | 0 |
| **Stress-70 protein, mitochondrial** | P48721 | 73858 | 6.00 | 3.8E-07 | 1 | 0.19 |  | 1 | 0.52 |  | 1 | 0.43 |  | 1 |
| **Thiosulfate sulfurtransferase** | P24329 | 33407 | 7.70 | 2.5E-09 | 2 | 11.71 |  | 1 | 6.56 |  | 1 | 8.75 |  | 1 |
| **Thy-1 membrane glycoprotein** | P01830 | 18172 | 9.40 | 4.0E-07 | 1 |  |  | 0 |  |  | 0 |  |  | 0 |
| **Thymosin beta-4** | P62329 | 5053 | 5.00 | 2.1E-05 | 2 | 6.49 | 4.69 | 2 | 2.63 | 0.33 | 2 | 4.65 | 3.36 | 2 |
| **Thyroid hormone receptor-associated protein 3** | Q5M7V8 | 108253 | 10.20 | 6.2E-05 | 1 |  |  | 0 |  |  | 0 |  |  | 0 |
| **Transaldolase** | Q9EQS0 | 37460 | 6.60 | 2.5E-03 | 2 | 8.68 | 11.81 | 2 | 0.46 | 0.29 | 2 | 1.64 | 1.23 | 2 |
| **Transcription elongation factor A protein 1** | Q4KLL0 | 33894 | 8.60 | 1.2E-06 | 1 | 4.41 |  | 1 | 4.07 |  | 1 | 4.99 |  | 1 |
| **Transgelin-3** | P37805 | 22501 | 6.80 | 2.5E-09 | 1 | 0.20 |  | 1 | 0.72 |  | 1 | 0.38 |  | 1 |
| **Transketolase** | P50137 | 67644 | 7.20 | 2.1E-05 | 4 | 3.98 | 0.00 | 2 | 5.36 | 3.32 | 2 | 5.60 | 6.47 | 3 |
| **Trifunctional enzyme subunit alpha, mitochondrial** | Q64428 | 82666 | 9.20 | 4.2E-05 | 1 |  |  | 0 |  |  | 0 |  |  | 0 |
| **Tropomyosin alpha-3 chain** | Q63610 | 29007 | 4.70 | 1.9E-05 | 5 | 0.48 | 0.05 | 3 | 3.86 | 6.99 | 4 | 6.76 | 7.77 | 5 |
| **Tropomyosin alpha-4 chain** | P09495 | 28510 | 4.70 | 6.3E-04 | 3 | 4.88 | 5.03 | 3 | 5.86 | 7.46 | 3 | 11.72 | 12.58 | 3 |
| **Tubulin alpha-1A chain** | P68370 | 50136 | 4.90 | 2.5E-09 | 1 | 0.75 |  | 1 | 10.38 |  | 1 | 3.23 |  | 1 |
| **Tubulin beta-5 chain** | P69897 | 49671 | 4.80 | 1.3E-07 | 1 |  |  | 0 |  |  | 0 |  |  | 0 |
| **Uteroglobin** | P17559 | 10449 | 4.90 | 8.6E-05 | 3 | 0.28 | 0.05 | 2 | 0.21 | 0.04 | 2 | 0.33 | 0.00 | 2 |
| **Vimentin** | P31000 | 53733 | 5.10 | 1.0E-09 | 10 | 6.11 | 8.60 | 9 | 4.71 | 6.46 | 9 | 5.41 | 6.06 | 9 |
| **Vinculin** | P85972 | 116616 | 5.80 | 4.2E-05 | 1 |  |  | 0 |  |  | 0 |  |  | 0 |

**Information on Supplemental Data:**

**Accession Number:** Swissprot/ Uniprot accession number. The purpose of accession numbers is to provide a stable way of identifying entries from database release to database release. It is sometimes necessary for reasons of consistency to change the names of the entries, for example, to ensure that related entries have similar names. However, an accession number is always conserved, and therefore allows unambiguous citation of entries (<http://web.expasy.org/docs/userman.html>) (accessed 4/9/2013).

**Expectation Value:** The probability value (p-value) is the probability an event will occur at random. The expectation value (e-value) is the expected number of times an event will occur at random from a given set of trials ( e-value = p-value * number of trials ). For mass spectrometry, an e-value is the number of times a given peptide score (or greater) will be achieved by incorrect matches from a database search. If a peptide assignment has an e-value of 0.01, then one would expect 1 peptide to match at random from a database 100 times in size.

E-values in Protein prospector are calculated by fitting the incorrect (null) results from a database search to a distribution and using this distribution to calculate a p-value (1,2)

For more information of Protein prospector’s calculation of expectation values please refer to <http://prospector.ucsf.edu/prospector/html/misc/publications/2006_ASMS_1.pdf> (accessed 4/9/2013)

**Total Peptides:** Total number of peptides identified for this protein

**Mean / Standard deviation (STD):** Mean result for quantitative comparison of NSNV versus SNV, NSV and SV using iTRAQ labeling.

**Peptides for Quantitation:** Number of peptides used for calculation of quantitative results.

**References for Supplement**:

1. Craig, R. and Beavis, R. C. (2004). “TANDEM: matching proteins with tandem mass spectra.” **Bionformatics 20**(9):1466-7.

2. Fenyo, D. and Beavis, R. C. (2003). “A Method for Assessing the Statistical Significance of MS Based Protein IDs Using General Scoring Schemes.” **Anal Chem 75**:768-774.
